# Wireless Miniature Magnetic Phase-Change Soft Actuators

**DOI:** 10.1002/adma.202204185

**Published:** 2022-08-17

**Authors:** Yichao Tang, Mingtong Li, Tianlu Wang, Xiaoguang Dong, Wenqi Hu, Metin Sitti

**Affiliations:** School of Mechanical Engineering Tongji University Shanghai 201804, China; Physical Intelligence Department Max Planck Institute for Intelligent Systems 70569, Stuttgart, Germany; Physical Intelligence Department Max Planck Institute for Intelligent Systems 70569, Stuttgart, Germany; Institute of Functional Nano & Soft Materials (FUNSOM) Jiangsu Key Laboratory for Carbon-Based Functional Materials & Devices Soochow University Suzhou, Jiangsu 215123, China; Physical Intelligence Department Max Planck Institute for Intelligent Systems 70569, Stuttgart, Germany; Institute for Biomedical Engineering ETH Zurich Zurich 8092, Switzerland; Physical Intelligence Department Max Planck Institute for Intelligent Systems 70569, Stuttgart, Germany; of Mechanical Engineering Vanderbilt University Nashville, TN 37215, USA; Vanderbilt Institute for Surgery and Engineering Vanderbilt University Nashville, TN 37215, USA; Physical Intelligence Department Max Planck Institute for Intelligent Systems 70569, Stuttgart, Germany; Physical Intelligence Department Max Planck Institute for Intelligent Systems 70569, Stuttgart, Germany; Institute for Biomedical Engineering ETH Zurich Zurich 8092, Switzerland; School of Medicine and College of Engineering Koç University Istanbul 34450, Turkey

**Keywords:** high work capacity, magnetic soft composites, miniature wireless soft devices, phase-change materials, programmable shape deformation

## Abstract

Wireless miniature soft actuators are promising for various potential high-impact applications in medical, robotic grippers, and artificial muscles. However, these miniature soft actuators are currently constrained by a small output force and low work capacity. To address such challenges, a miniature magnetic phase-change soft composite actuator is reported. This soft actuator exhibits an expanding deformation and enables up to a 70 N output force and 175.2 J g^−1^ work capacity under remote magnetic radio frequency heating, which are 10^6^–10^7^ times that of traditional magnetic soft actuators. To demonstrate its capabilities, a wireless soft robotic device is first designed that can withstand 0.24 m s^−1^ fluid flows in an artery phantom. By integrating it with a thermally-responsive shape-memory polymer and bistable metamaterial sleeve, a wireless reversible bistable stent is designed toward future potential angioplasty applications. Moreover, it can additionally locomote inside and jump out of granular media. At last, the phase-change actuator can realize programmable bending deformations when a specifically designed magnetization profile is encoded, enhancing its shape-programming capability. Such a miniature soft actuator provides an approach to enhance the mechanical output and versatility of magnetic soft robots and devices, extending their medical and other potential applications.

## 1 Introduction

Wireless miniature soft actuators can enable devices that operate in enclosed confined spaces, making them appealing for microfabrication,^[1]^ medical,^[2]^ and other real-world applications.^[3]^ However, despite these advances, it is still challenging for these actuators to achieve a large output force and high weight-normalized work capacity (referred to as “work capacity” here-after). ^[4]^ This is because the softness of the constituent materials and the limited volume are hard to store and release high mechanical energy.^[2d,5]^ Currently, most miniature soft actuators demonstrate a relatively low work capacity in the range of 10^–3^ to 10^2^ J kg^−1^ (Figure S1, Supporting Information),^[3b,6]^ which does not allow them to be used in potential medical devices, manipulation and other applications requiring high work capacity.^[7]^ Furthermore, the maximum output forces of the existing magnetic soft actuators are ≈60 μN. However, many medical procedures, such as stenting,^[8]^ require the device with an output force of over 1 N, which is ≈10^4^ times that of the maximum output force of magnetic soft actuators.

Soft pneumatic actuators have simultaneously offered high mechanical performance and compliance, enabling a wide range of applications in powerful manipulations.^[9]^ Specifically, despite a small Young’s modulus of ≈10 kPa, these actuators can deliver a high work capacity (9 J g^−1^), outperforming most of the reported actuators by a factor of ≈10^1^–10^3^. Although shape-memory alloy has a similar work capacity, it is 10^6^ times stiffer (Figure S1, Supporting Information).^[10]^ However, pneumatic actuation suffers from the accompanied compressors, valves, pumps, and connecting pipes.^[9b,11]^ This makes the whole system bulky. To address these issues, recent works have proposed actuators based on wirelessly induced liquid-to-gas phase change via magnetic radio frequency (RF) heating.^[12]^ This improvement removes the external connection to a power resource, battery, and pump. In addition, it can deliver a similar actuation strain and work capacity to biological muscles. However, the existing phase-change actuators, e.g., with a diameter of 16.9 mm and length of 63 mm,^[12,13]^ are still too large for potential miniature robotic and device applications,^[14]^ or unable to deploy with wireless actuation.^[15]^ It would be beneficial if the actuator could wirelessly control the device’s rigid-body motion, besides its inflation and deflation, toward minimally invasive medical applications.^[2b,h,16]^

**Figure 1:**
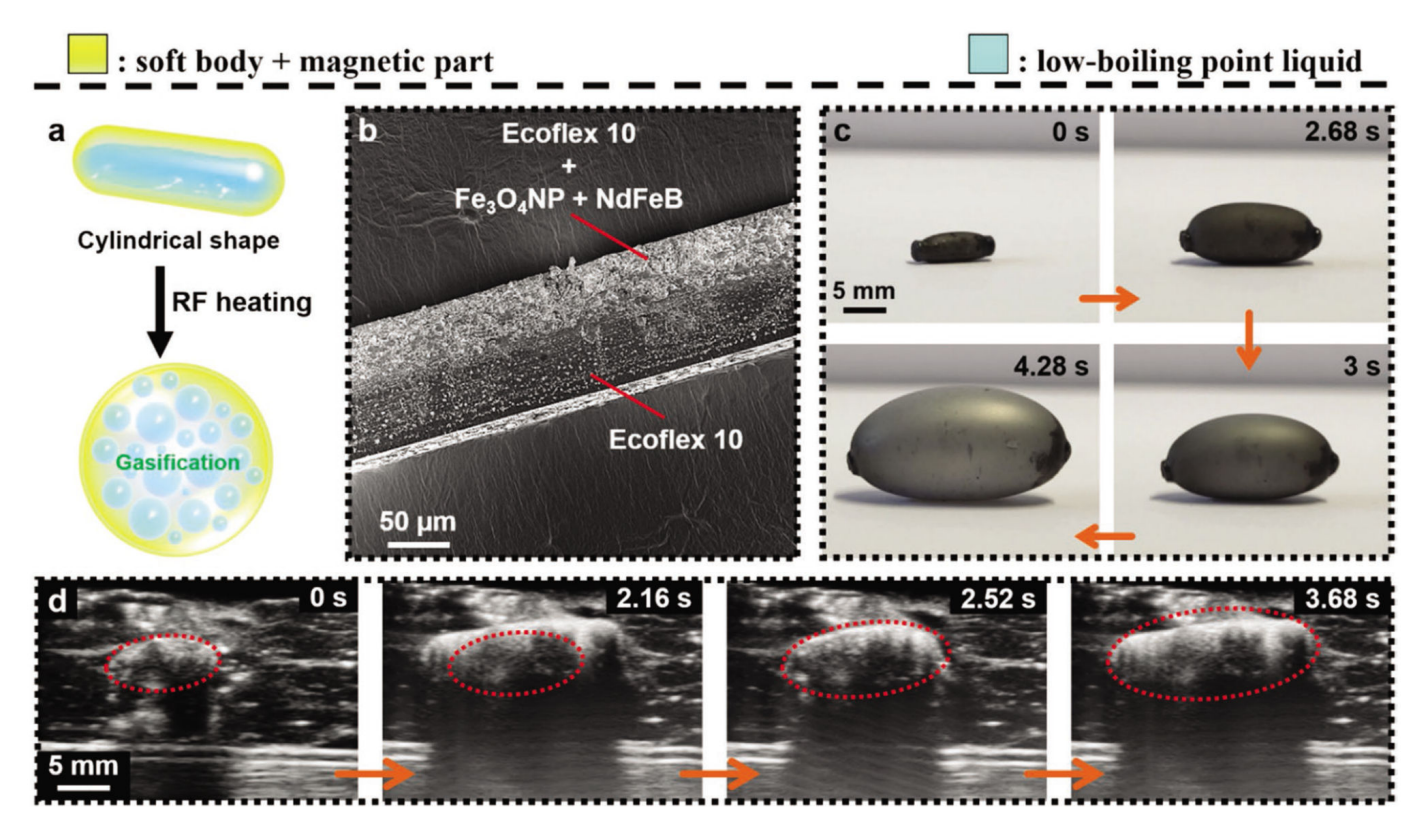
Phase-change magnetic soft actuator with inflation behavior. a) Schematic showing the inflation process of the cylindrical phase-change actuator under magnetic-RF heating. b) Scanning electron microscope (SEM) image of the composed double-layer film for the actuator. c) Experimental camera video snapshots exhibit the corresponding inflation process of the actuator under magnetic-RF heating. d) Ultrasound imaging video snapshots exhibiting the inflation process of the cylindrical actuator inside a porcine coronary artery *ex-vivo*. The images in (c) and (d) are obtained from Movies S1 and S3 (Supporting Information), respectively.

To tackle these challenges, here we propose a balloon-shaped inflatable wireless miniature soft actuator using a magnetic soft composite, which is composed of silicone rubber (Ecoflex) with embedded hard-magnetic NdFeB microparticles and iron-oxide Fe_3_O_4_ nanoparticles (Fe_3_O_4_NPs). Moreover, the balloon is filled with a low boiling point liquid (Novec 7000, the boiling point is ≈34 °C, which is biocompatible Figure 1a).^[17]^ The NdFeB micro-particles enable it to be actuated via the external magnetic field. Also, remote magnetic-RF field-based heating of Fe_3_O_4_NPs enables liquid phase change-based inflation that can exhibit a boost in the output force (70 N) and work capacity (175.2 J g^−1^). The proposed phase change actuator is a combination of magnetic and pneumatic soft actuation methods. This new design takes the complementary advantages of these two methods. In Figure S1 (Supporting Information), we have compared the work capacity with other different soft actuators. Specifically, with a similar size, the proposed phase-change actuator is 10^7^ times larger for the output force and 10^6^–10^7^ times higher in work capacity than that of magnetic soft composite actuators.^[6f]^ Furthermore, when compared with typical phase-change soft actuators in the literature, as shown in Table S1 (Supporting Information), the proposed actuator has a smaller size, a larger actuation strain, and a higher work density.

First, we specifically characterize the inflation behavior of this phase-change actuator. Then, we explore the design principle, i.e., material and geometrical parameters, of this phase-change actuator to achieve superior mechanical performance. With this knowledge, we develop a generalized theoretical model as the reference to guide the design and aid in understanding its nonlinear behavior, including the relationship among design parameters (e.g., materials modulus and thickness), control inputs (internal pressure of the actuator), and outputs (including stroke, force, and work capacity). Next, we showcase this actuator to be a soft device prototype toward angioplasty applications in the future and explore widening the functions of such an actuator for biomedical applications by integrating it with a thermally responsive shape-memory composite and bistable metamaterial sleeve. This bistable structure endows the phase-change actuator with reversible shape changes and self-locking capabilities. We demonstrate this device to have a controllable open and close capability by adjusting the power of the remote magnetic-RF heating input. Furthermore, its capability to move inside and jump out of the granular media is demonstrated. At last, this phase-change actuator is encoded with a specifically designed magnetization profile to realize deformations induced by internal magnetic torques due to applied external magnetic fields.^[18]^ Besides boosting the force output, this combination of magnetic soft material and phase-change material adds programmable bending deformation capability (in addition to programmable inflation and deflation) to the actuator, further enriching its potential applications in soft robotics and biomedical engineering.

## 2 Results and Discussion

### 2.1 Soft Actuator Design Methodology

First, we design a cylindrical-shaped phase-change actuator to load the low-boiling point liquid (Figure 1a). The fabrication process is shown in Figure S2 (Supporting Information). Firstly the rectangular double-layer film is wrapped onto the surface of a glass rod to construct the cylindrical shape. Next, the edges and two ends are sealed up with a pure Ecoflex elastomer. As shown in Figure 1b and Figure S3 (Supporting Information), the inner layer of the film is composed of an Ecoflex elastomer matrix doped with Fe_3_O_4_NPs and NdFeB micro-particles. The function of Fe_3_O_4_NPs is to heat the actuator under a magnetic-RF heating system based on the magneto-thermal effect.^[12]^ The NdFeB microparticles induce magnetic torque or force to wirelessly and precisely control the actuator. The outer layer is constructed by a pure Ecoflex elastomer to improve the airtightness of the actuator. After the sealing process, we programmed the magnetic moment to be along the axial direction of the actuator with a uniform magnetic field of 1.8 T. Finally, the low-boiling point liquid was injected into the interior of the actuator to obtain a cylindrical-shaped phase-change actuator. As the mechanism shown in Figure 1c and Movie S1 (Supporting Information), under the magnetic-RF heating, the internal layer of the actuator can be heated, causing the entire actuator temperature to increase. Therefore, the internal low boiling point liquid vaporizes, eventually inducing the inflation of the actuator. As can be seen from Figure S4 (Supporting Information) and Movie S2 (Supporting Information), the actuator can continuously operate for at least 3 min without or with a small volume change. In addition, we put the actuator into the porcine coronary artery, as shown in Figure 1d and Movie S3 (Supporting Information). Through ultrasound imaging (Vevo 3100), we can see that the inflation force of the actuator is large enough to withstand the porcine coronary artery (0.19–1.09 N is required).^[19]^ Such behavior could be used to treat vascular blockage, embolism, and other diseases in the future.^[20]^

**Figure 2:**
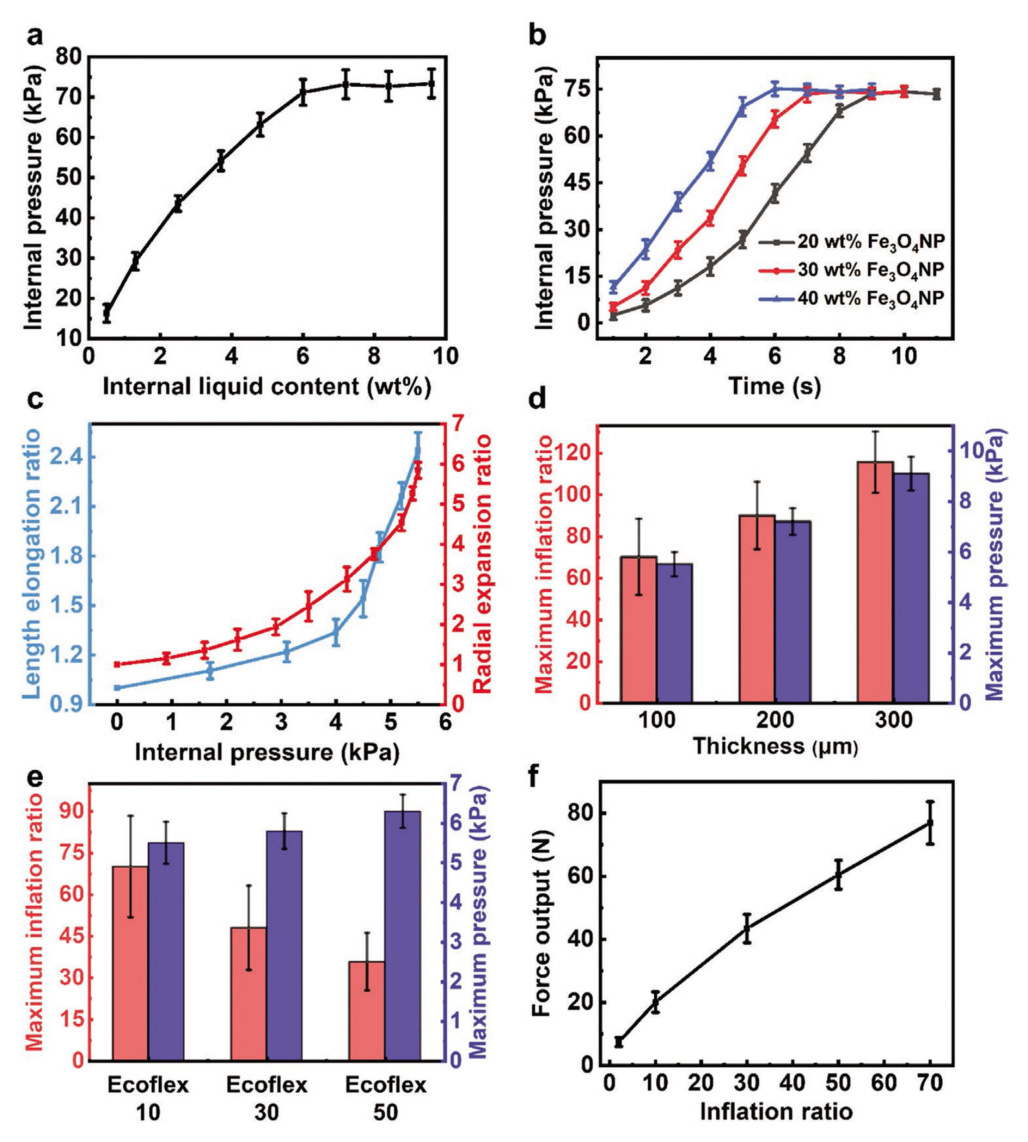
Characterization and optimization of the magnetic phase-change soft actuator. a) The maximum internal pressure of the phase-change actuator with a fixed 70.8 mm^3^ volume under magnetic-RF heating as a function of the internal low-boiling point liquid content. b) The internal pressure of the actuator under magnetic-RF heating with different weight ratios of the Fe_3_O_4_NPs content. Note that the actuator volume is fixed to 70.8 mm^3^. c) The body length elongation ratio and radial expansion ratio of the actuator as a function of the inner pressure under magnetic-RF heating. The actuator’s maximum inflation ratio and internal pressure with different d) skin thicknesses and e) moduli of the composed materials in the inflation process. f) The calculated output forces under different volume inflation ratios. The data are presented as mean values ± standard deviation for 3 times measured.

Then we study the methodology to optimize the proposed actuator to achieve better performance. First, we look into the influence of the low-boiling point liquid content and the Fe_3_O_4_NPs content on the internal pressure of the actuator under magnetic-RF heating. In this process, we fix the magnetic phase change actuator into a tube with a volume of 70.8 mm^3^ to eliminate the effect of volume inflation. We use Ecoflex 10 with 100 μm thickness to construct the actuator in this experiment. As a result, increasing the liquid content can achieve higher internal pressures. The maximum internal pressure can reach up to 73.2 kPa when the liquid content is higher than 6.4 wt.% (Figure 2a). For the influence of Fe_3_O_4_NPs content, as shown in Figure 2b, increasing the Fe_3_O_4_NPs content can achieve higher heating efficiency, making the pressure inside the actuator increase faster. However, although the heating efficiency of the actuator changes with the different Fe_3_O_4_NPs content, the peak pressure is similar, about 73.2 kPa. This is because the heat transfer between the actuator and the environment prevents further temperature increases.

Second, we study the impact of the material modulus and thickness on stroke, i.e., actuator inflation and deflation. In the inflation process, there is a competition between the inflation energy generated by the inner pressure change (*U*_pressure_), and the strain energy in the composed soft elastomer (*U*_actuator_). The total potential energy (*U*_total_) is the sum of these two energies:^[21]^

(1) $$U_{\text{total}}=U_{\text{actuator}}+U_{\text{pressure}}$$

Upon inflation, the cylindrical actuator expands along in both axial and radial directions. Its theoretical values of radius (*r*) and length (*L*) at a given inner pressure (*P*) can be obtained by minimizing the total potential energy (see details in Experimental Section “Model for a single-channel pneumatic actuator”). According to Equations 1 and 6, the results shown in Figure S5 (Supporting Information) indicate that both *r* and *L* increase monotonically with *P*, i.e., the higher the inner pressure, the larger inflation achieved after actuation. To validate the model, we experimentally examine the inflation of the actuator. The schematic and the setup to measure the internal pressure are shown in Figure S6 (Supporting Information). As shown in Figure 2c and Figure S7 (Supporting Information), the results indicate that, even with a small inner pressure at 5.5 kPa, the actuator elongates by 5.8 and 2.4 times along the radial and axial direction, respectively, resulting in 71.1 times volumetric inflation. This is because of the small strain energy of the composed materials. In addition, according to Equation 1, to further increase the inflation stroke, the strain energy of the composed materials needs to be reduced. To evaluate the influence of the composed materials’ mechanical performance on the strain energy, we study the internal pressure and volume inflation ratio of the actuator as a function of the constructed materials with different moduli and thicknesses, respectively (Figure 2d,e). The results indicate that the softer actuator (smaller modulus) takes a larger volume inflation ratio and requires lower internal pressure. Although the actuator with thicker skin has a larger volume inflation ratio, the required internal pressure is higher. Therefore, either softening the actuator or decreasing the actuator thickness can reduce the strain energy of the actuator. But the material should not be too soft; otherwise, it will cause the liquid in the cavity to release. As a result, according to these experimental results, it is best to choose the Ecoflex-10 as the base material with 100 μm thickness.

With a knowledge of the internal pressure and inflation stroke, we can evaluate the output force and work capacity. The output force of the actuator can be defined as

(2) $$F=\left(P-P_{\text{c}}\right)\times 2\pi RL$$

where *R*, *P* and *L* indicate the radius, internal pressure and the length of the actuator after inflation, respectively; *P*_c_ is the internal threshold pressure when the actuator reaches the corresponding radius and length. Here we first confine the actuator with no inflation under magnetic-RF heating by putting the actuator into a designed glass tube (i.e., *R* = 1.5 mm, *P*_c_ = 0). Therefore, the generated internal pressure of the actuator directly exerts the force on the glass tube, resulting in the energy input barely transiting into strain energy in soft material but mainly converting into energy output. We plot the output force of the actuator with respect to the inner pressure in Figure S8 (Supporting Information). It shows *F* increases gradually and monotonically with heating times and the maximum force can reach 8.1 N. Furthermore, we study the output force versus the volume inflation ratio by confining the actuator’s inflation radius and length with different values. As shown in Figure 2f, the calculated output force increases with increasing the designed inflation ratio and the maximum output force can reach 76.9 N when the inflation ratio is ≈70. Moreover, we also plot the output force with different material compositions and thicknesses in Figure S9 (Supporting Information). In this experiment, the volume inflation ratio is fixed to ≈10. It shows that softer and thinner actuators exhibit a larger output force. This trend, together with the result from Figure 2d,e, suggests that decreasing the modulus and thickness of the actuator provides more mechanical output, showing its unique capability of leveraging compliant materials for powerful mechanical outputs, i.e., inflation.

We assume the actuator is subjected to a uniform external pressure, *P*_ext_, to calculate the work capacity during the whole inflation process. The corresponding work capacity, *w*, is expressed as

(3) $$w=\frac{P_{\text{ext}}\Delta V}{m}$$

where Δ*V* is the volume change, and *m* is the mass of the actuator. Figure S10 (Supporting Information) plots the theoretical radial inflation of the actuator as a function of *P*_ext_ for actuators with different inner pressure, *P*. For each actuator, its stroke increases with inner pressure. However, it decreases with the external load, and the maximum stroke is realized for the lowest external pressure. From Equation 3, we can predict the work capacity resulting from external pressure and inner pressure (Figure S11, Supporting Information), where all actuators show similar “n”-shape curves,^[22]^ i.e., *w* first increases steeply with *P*_ext_, then approaches the peak followed by a gradually drop. The maximum work capacity is 175.2 J g^−1^ under *P*_ext_ = 28 kPa. This work capacity is ≈4600 times that of natural muscle, and 3300% stronger than the benchmarking high-energy twisted-and-coiled muscle.^[23]^ Figure S11 (Supporting Information) also shows that work capacity increases drastically with the internal pressure of the actuator. The peak work capacity can be manipulated from 10.1 to 175.2 J g^−1^ by adjusting the inner pressure from 35 to 75 kPa, showing a nonlinear 17.5 times amplification by only doubling the inner pressure. This could be attributed that the proposed phase-change actuator being a kind of pneumatic actuator, which is a highly nonlinear system. This nonlinear performance is mainly attributed to the competition between the volume expansion of the actuator and the accumulated strain energy in the actuator skin.^[24]^ In this sense, the inner pressure plays a dominant role in determining the upper limit of the mechanical performance of this phase-change system. Additionally, we have studied the influence of the moduli and thickness of the actuator skin on the work capacity. As shown in Figure S12 (Supporting Information), the work capacity decreases with the increasing moduli and thickness of the composed skin. This is because the strain energy (i.e., *U*_actuator_ in Equation 1) of the composed soft elastomer increases with these two parameters in the inflation process, thus reducing the work capacity.

### 2.2 Wirelessly Activated Balloon Toward Angioplasty

**Figure 3:**
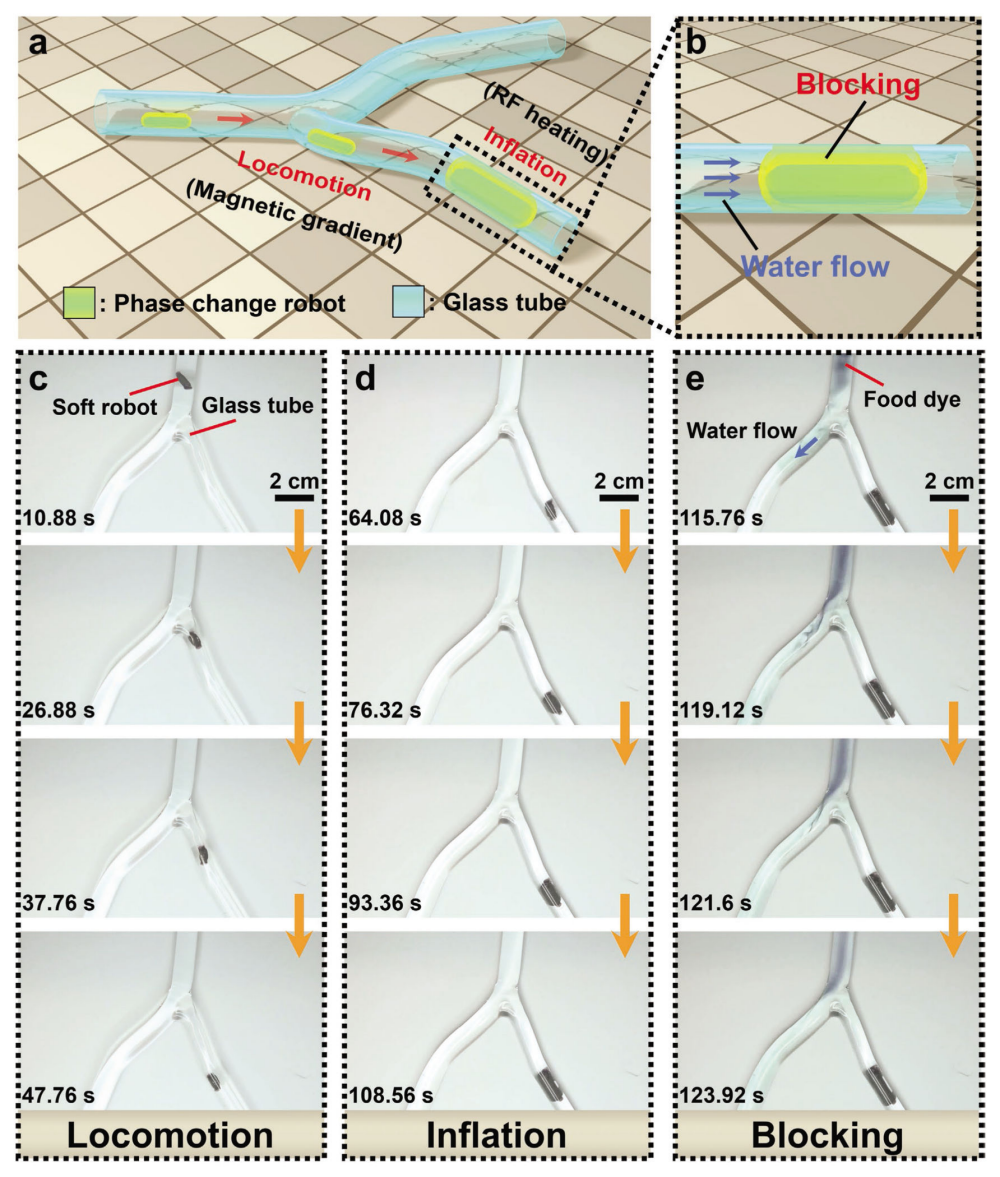
Actively controlled soft inflating balloon toward future angioplasty applications. Schematic illustrates a) the locomotion and inflation, and b) blocking process of the magnetic phase-change actuator under a rotating magnetic field and magnetic-RF heating, respectively. Note, the amplitude and frequency of the rotating magnetic field are about 40 mT and 0.5–2 Hz, respectively. A series of pictures illustrate the corresponding c) locomotion, d) inflation, and e) blocking process in the “Y”-shaped water full-filled glass tube. The images in (c)–(e) are obtained from Movie S4 (Supporting Information).

Coronary artery diseases affect millions of people in the world. This disease is an atherosclerosis process, which will build up the plaque and cause stenosis and occlusion of arteries.^[25]^ One efficient option to treat this disease is using balloon angioplasty and deploying a stent. Balloon angioplasty utilizes balloon inflation to open the narrowed artery, which is driven by a catheter.^[26]^ To explore an untethered balloon angioplasty application in the future and potentially reduce incision damage, here we demonstrate the proposed magnetic phase-change actuator as an untethered miniature soft device toward angioplasty inside a tubular structure (Figure 3a). Specifically, we showcase the fluid clogging of this actuator in a “Y”-shaped vascular phantom (Figure 3a,b; Movie S4, Supporting Information). First, we apply a rotating magnetic field on the soft device so that it can rotate while overcoming the kinetic frictional resistance from the glass wall. Meanwhile, the soft device is further pulled forward by a magnetic spatial gradient pulling force (Figure 3c). When the actuator is reached the target location, the rotating magnetic field is switched off and the magnetic-RF heating is activated to trigger the inflation of the magnetic phase-change soft actuator (Figure 3d). Then, we use a peristaltic pump to generate the water flow condition. As shown in Figure 3e and Movie S4 (Supporting Information), this device can maintain its position despite a continuous strong pulsatile water flow (average speed: 0.24 m s^−1^) after it is deployed. As a reference, this water flow is higher than the maximum blood flow velocity in a vascular artery (0.049–0.19 m s^−1^),^[27]^ showing that the device could be potentially used in future occlusion, e.g., embolization applications also.

**Figure 4:**
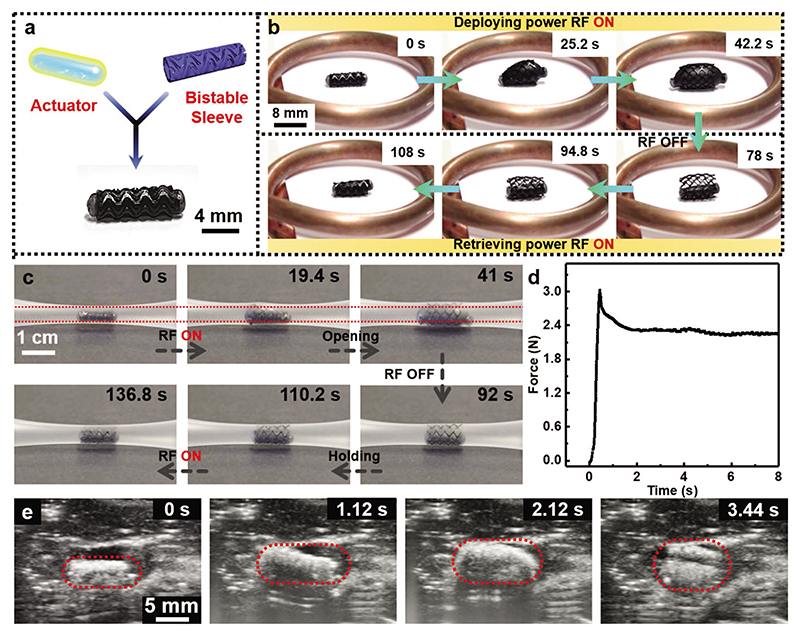
Proof-of-concept demonstration of the reversible bistable stent application. a) Schematic and camera image showing the design of the reversible stent. b) A series of camera images showing the operating process of the reversible stent with different power inputs of the magnetic-RF heating. c) The images exhibit the “open” and “close” process of the reversible stent in the Ecoflex soft tube and d) the corresponding stenting force in this process. e) The ultrasound images demonstrate that the reversible stent opens inside the porcine coronary artery *ex-vivo*. The images shown in (b), (c), and (e) are obtained from Movies S5, S6, and S7 (Supporting Information), respectively.

A stent is often placed in the balloon angioplasty procedure to keep the artery open after deflating and removing the balloon.^[26b]^ We explore combining the actuator with elastic instability and shape memory effect to realize a controllable and reversible stenting function. We demonstrate a millimeter-scale magnetic robot capable of large force inflation (≈3 N), self-locking, and shape memory effect for future reversible stent application purposes in a tubular phantom. Figure 4a shows the schematic of the robot design. It is constructed by encapsulating a magnetic phase-change actuator in a bistable metamaterial sleeve. The bistable sleeve is made of a thermally responsive SMP and also embedded with Fe_3_O_4_NPs to enable remote control by magnetic-RF heating (the fabrication process of the bistable shape memory metamaterial sleeve is shown in the Experimental Section).

The bistability-enabled self-locking can realize a long-lasting operation of the robot at the target tissue without continuously applying the input signal. As experimentally illustrated in Figure 4b and Movie S5 (Supporting Information), the working principle follows three steps. First (Figure 4b, 0 to 42.2 s), we activate the magnetic-RF heating system at a deploying power input (P2, the parameters of the power input shown in Table S2, Supporting Information) to trigger the inflation of the magnetic phase-change actuator. Please note that this input energy and others mentioned hereafter are displayed value on the equipment but not measured at the operation site. This inflation deforms the bistable sleeve from its initial contracted stable state to the expanded stable state, resulting in a 2.3 times radial inflation so that the robot anchors. Second (Figure 4b, 42.2 to 78 s), we switch off the magnetic-RF field resulting in the phase change actuator deflating, whereas the bistable sleeve maintains its open stable state. Third (Figure 4b, 78 to 108 s), we apply the magnetic-RF field at a retrieving power input (P1). In this way, only the shape memory skin is activated and reverts to its original state. Additionally, as shown in Figure 4c and Movie S6 (Supporting Information), we demonstrate that the stent can still realize this reversible stenting behavior in a controllable manner inside a soft tube. The detailed working principle of the device is shown in the Experimental Section, “Principle of the reversible bistable stent”. The anchoring force of this bistable sleeve at the expanded state is measured to be 3 N (Figure 4d), which is higher than the performance of a vascular stent with a similar size (0.25 N).^[28]^ As shown in Figure 4e and Movie S7 (Supporting Information), the stent can hold up a porcine coronary artery and is anchored due to its bistability. This allows our design to be used as a future untethered soft stenting robot with re-positioning capability.

### 2.3 Soft-Bodied Locomotion Inside Granular Media

**Figure 5:**
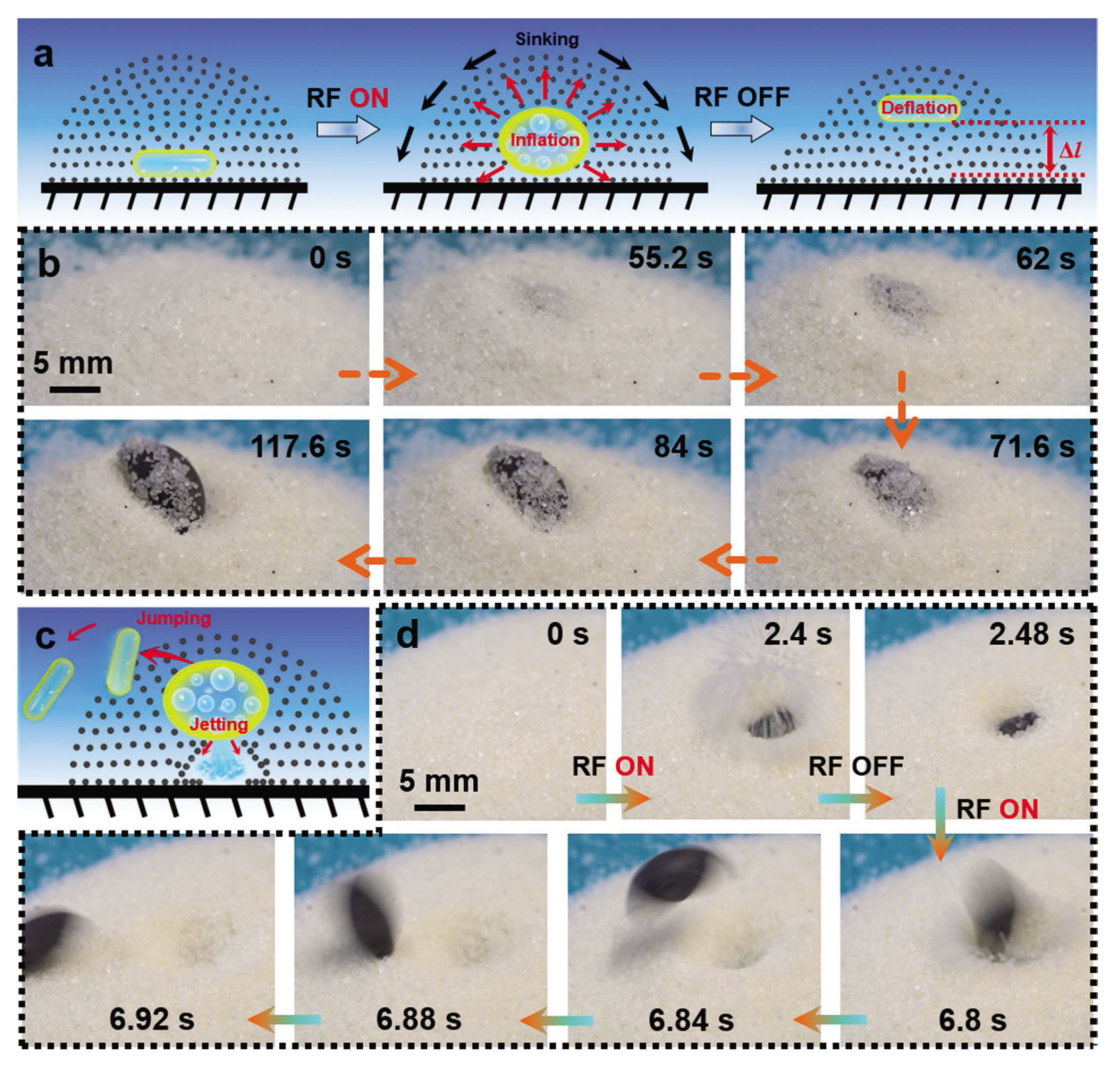
Soft-bodied locomotion capability of the proposed actuator inside granular media. a) The Schematic illustrates the vertical lifting process of the magnetic phase-change actuator inside a granular media by turning the magnetic-RF heating system on and off at regular intervals. b) The corresponding camera images of this lifting process. c) The Schematic shows the explosion and jumping behavior of the magnetic phase-change actuator in granular media under magnetic-RF heating (P3 power input). d) The corresponding images of the explosion and jumping process. The images shown in (b) and (c) are obtained from Movies S8 and S9 (Supporting Information), respectively.

A soft body with large and large degrees-of-freedom deformations enables robust and multimodal locomotion modalities in complex terrains and media.^[18a,29]^ While many studies include soft-bodied locomotion in fluids and air, only a few studies have tackled soft-bodied locomotion in granular media so far.^[29g,h,30]^ Unlike in air or fluidic media, the motion of the wireless soft robots inside the subterranean granular media is hard to realize due to higher resistance forces.^[29a]^ Here, we explore the proof-of-concept locomotion capability of the proposed actuator inside granular media, as it has a large deformation strain and output force. As shown in Figure 5a,b and Movie S8 (Supporting Information), the actuator is placed inside the granular medium, which consists of commercial salt particles with an average 1.2 mm diameter, at the beginning. By turning the magnetic-RF heating system on and off at regular intervals, the actuator expands and contracts sequentially, leading to the actuator being lifted from the inside of the granular media to the surface, due to the gradient in the lithostatic pressure.^[30a]^ The sand particles sink downward due to gravity during the actuator inflation and deflation process. As a result, there is a relative movement between the actuator and the sand particles, causing the actuator to move toward the surface (Figure 5a).

The soft balloon can also exhibit a jumping phenomenon. After applying power input energy (P3) to the magnetic-RF heating system, the actuator exhibits a sudden actuation similar to an explosion. As shown in Figure 5c,d and Movie S9 (Supporting Information), this actuator can jump out of the granular media due to such an explosion. This phenomenon is caused by the excessive pressure generated inside the actuator under very high magnetic-RF heating power, leading to the rupture of the silicone elastomer skin, and rapidly releasing the internal gas. In addition, the small ruptured hole seals itself after every jump as the Ecoflex skin is viscoelastic.^[31]^ Consequently, the actuator can explode multiple times before depleting the phase-change liquid inside. For the current actuator, the explosion and jumping behavior can be repeated about ten times. Furthermore, the proposed actuator can locomote inside the granular media composed of sand and pebbles, as shown in Figure S13 (Supporting Information) and Movie S10 (Supporting Information). This further demonstrates the potential application in a more realistic environment.

### 2.4 Bimodal Phase-Change and Magnetic Soft Actuation

**Figure 6:**
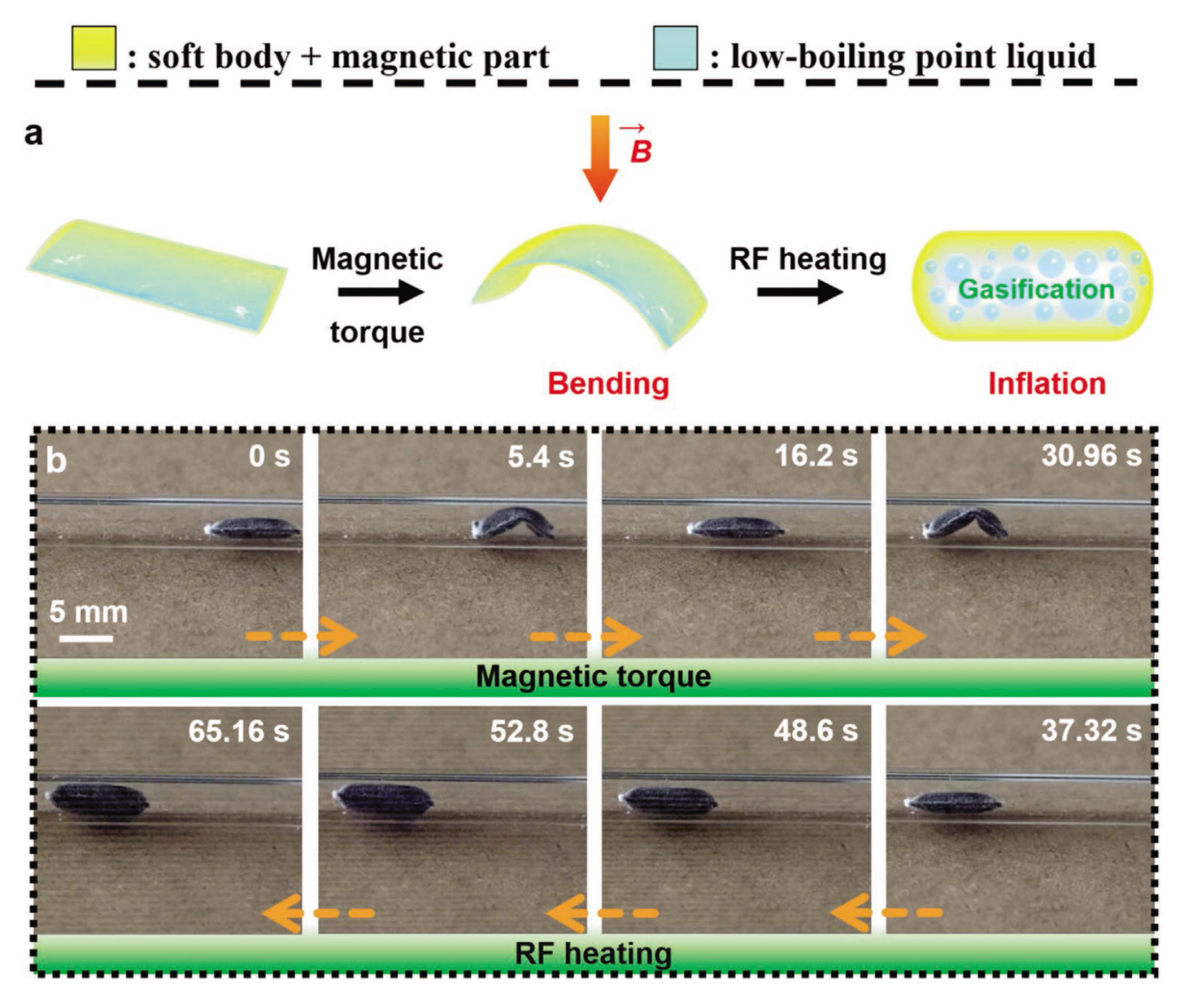
Sheet-shaped magnetic phase-change soft actuator with walking and expanding behavior. a) The schematic illustrates the bending and expanding behaviors of the actuator under magnetic torques and magnetic-RF heating, respectively. b) A series of images showing the walking and inflation behavior of the magnetic phase-change soft robot inside a glass tube under magnetic torques and magnetic-RF heating, respectively. These images are obtained from Movie S11 (Supporting Information).

Compared with other miniature soft actuators driven by electricity,^[29f,32]^ chemical,^[33]^ optical,^[34]^ and other stimuli,^[35]^ magnetic actuation is fast, safe, and precise, offering much better versatility and controllability for fixed-volume workspaces.^[18a]^ Although locomotion of magnetic soft robots has been previously reported, they mainly employ bending body deformations for inducing different locomotion gaits.^[9a,36]^ In addition, compared with other reported actuators (Figure S1, Supporting Information), magnetic soft composite actuators at the millimeter-scale has a low work capacity of ≈2 × 10^–4^ kJ kg^−1^ with a small concomitant reaction force of ≈60 μN. Therefore, we combine phase-change and magnetic actuation to create a sheet-shaped magnetic phase-change soft robot (Figure 6). This robot is prepared by laminating two rectangular double-layer magnetic films with the edges of the films bonded together to form a rectangular channel between the two films. After finishing the packaging process, we program a sinusoidal magnetization profile into the robot body by rolling it onto the surface of a glass rod and magnetizing it with a uniform magnetic field of 1.8 T (Figure S14, Supporting Information).^[18a]^ Next, we inject the robot with the low-boiling point liquid as fuel. After infusion, the robot becomes thicker but still holds the sheet shape. Therefore, as shown in Figure 6a,b and Movie S11 (Supporting Information), the magnetic phase-change actuator can thus be controlled to crawl inside a tubular channel. In this process, based on the magnetic torque and gradient,^[37]^ we use an external permanent NdFeB magnet to apply the external magnetic field and induce the body shape deformation. As shown in Figure 6b and Movie S11 (Supporting Information), when the actuator is steered to the target location, we use magnetic-RF heating to trigger the robot inflation, eventually causing the robot to inflate and block the pipe. This interchange between a sheet- and a balloon-shape with a 64.3 times volume change shown here is drastically different from the previously shown shape-morphing studies, and could potentially enable various high force and work capacity-output applications that are not possible to realize by the previously reported magnetic soft robots. This magnetic shape programming capability can potentially enable the robot to have additional diverse shape deformations that can be utilized for navigating through more complex environments.

## 3 Conclusions

Several points need to be addressed in future work to broaden the functionalities of this magnetic phase-change soft actuator. 1. The actuation temperature of the proposed actuator is ≈50 °C. It is essential to regulate the actuation temperature to adapt to physiologically relevant conditions. One option for reducing the actuation temperature is to decrease the modulus and thickness of the composed materials while maintaining airtightness. The other way is to use adaptive surfactants (such as FC-4430, 3M Company) to treat the Novec liquid to increase the produced gas, thus increasing the internal pressure at the same temperature and decreasing the response temperature. 2. Since the proposed actuator is a complex system in the inflation process, many factors can affect the relationship between internal pressure and temperature. Such as the nonlinear elasticity and inflation ratio of the composed elastomer; thermal transfer between the Fe_3_O_4_NPs, internal liquid, gas, and the elastomer skin; temperature distribution between the internal liquid and gas; and the changing magneto-thermal properties when the actuator inflates. It is worth extensive investigation in the future. 3. Although this work mainly focused on short-term applications, it is essential to further enhance the airtightness of the actuator for long-term applications, where two strategies can be performed in the future. First, we can select a material with even low permeability, such as natural rubber, to enhance the airtightness.^[38]^ Second, we can mix the Ecoflex silicone rubber with an urethane rubber to reduce its permeability.^[39]^ 4. This phase-change actuator is thermo-responsive, suffering from a low cooling response due to the slow thermal diffusion process. Especially, we add an extra protective Ecoflex layer on the outer surface of the actuator to prevent leakage. This insulation layer increases the heat diffusion path, leading to a longer cooling time. To further decrease the deflation time, one possible way is to use materials with high thermal conductivity to construct the protective skin. For example, in nature, the spider silk of Nephila clavipes spiders has an ultrahigh thermal conductivity up to 416 W mK^−1^, which is 2000 times higher than that of Ecoflex rubber.^[40]^ Also, the high thermal conductivity polymer can be designed with ordered structures, including controlled polymer chain orientations and the self-assembly of monolayers.^[41]^ 5. It is indeed possible to reduce the power input of the current design. The frequency of the current magnetic-RF heater is fixed at 377 kHz. It cannot be freely changed and therefore not optimized to match the resonant frequency of the Fe_3_O_4_NP. In the future, we can improve the heating efficiency by using a customized RF generation system whose output frequency can be tuned to resolve such issue.^[42]^ Moreover, the magnetic-RF induced thermal properties of nanoparticles highly depends on the oxide phase, morphology, shape and size of the Fe_3_O_4_NP.^[43]^ In the future, nanoparticles that can induce more efficient RF heating can be used. 6. The proposed soft actuator can achieve up to 10 jumpings. However, there are many factors that can affect the jumping performance. This includes the amount of liquid remaining inside the actuator, the elastic properties of the composite skin, the jetting location on the actuator skin, the size of the jetting hole, and the distribution of the sand surrounding the actuator. Therefore, after optimizing these parameters, a more robust and repeatable jumping can be achieved. 7. More complex morphology designs can enable a broader range of advanced functionalities. For example, we could build arbitrary topologies using the emerging voxel-based fabrication approach,^[44]^ where some voxels can be made of our proposed soft actuator. In this way, highly customized robots tailored for specific tasks with high mechanical performance could be developed.

## 4 Experimental Section

### Fabrication of the Magnetic Phase-Change Soft Actuators

First, 1 g of Ecoflex elastomer (10, 30, 50, Smooth-On Inc.) component A and component B were taken, and stirred and mixed for 1 min. Then, 2 g NdFeB microparticles (Magnequench, average diameter: 5 μm) and 1 g Fe_3_O_4_NPs (50–100 nm average diameter, Sigma–Aldrich) were added, stirred for 2 min, and mixed well. Next, the above-mentioned mixed polymer elastomer was put into a desiccator for degassing for 10 min. A 50 mm × 50 mm glass slide was prepared and placed on the spin coater, then the degassed elastomer composite material was poured onto the glass slide and spin-coated at 500 rpm for 3 min. Next, the spin-coated glass sheet was put into an oven (60 °C, Vacutherm, Thermo Scientific) to cure the polymer elastomer for 2 h, as the first layer of elastomer. Then 1 g of Ecoflex component A and component B were taken, respectively, stirred and mixed evenly and degassed, then poured on the cured polymer elastomer as the second layer. After performing the same spin coating operation, it was also placed in a 60 °C oven to cure for 2 h. Then a laser cutter (LPKF ProtoLaser U3) was used to cut the cured double-layer polymer elastomer into the desired shape. Next, the rectangular double-layer film was wrapped onto a glass rod to construct a cylindrical-shaped actuator. For the sheet-shaped actuator, two pieces of rectangular double-layer polymer elastomers were taken to contact with each other. The edges were sealed up with the mixture of Ecoflex component A and component B (1:1 mass ratio) to form a tube structure. Then, two 1.5 mm stoppers, composed of pure Ecoflex elastomer, were packaged on the two ends of the above tube structure to hold the shape of the actuator. After solidifying, a cavity structure was obtained. Next, the prepared cavity structure was magnetized in a 1.8 T uniform magnetic field using a vibrating sample magnetometer (EZ7, Microsense), and the magnetic field direction of the magnetic material in the actuator was programmed. Then, a syringe was used to inject Novec 7000 liquid (0.1 mL Sigma–Aldrich) from the stopper side into the prepared cavity structure. Before injecting, the air inside the actuator was sucked out to ensure more liquid could be injected. Finally, after sealing the inserted wound one more time to keep the airtightness, the final magnetically driven phase change actuator could be obtained.

### Thermal Characterization of the Magnetic Phase-Change Soft Actuator

The temperature of the actuator was measured when actuated by using a FLIR-A300 camera (FLIR Systems Inc.). The results were shown in Figure S15 (Supporting Information). It indicated that the actuation temperature was ≈50 °C, higher than the boiling point of the internal Novec 7000. This was because the high internal pressure increased the boiling point of the internal liquid, leading to a higher response temperature. The maximum actuation temperature was ≈52.8 °C. The relation between the internal pressure and temperature was shown in Figure S16 (Supporting Information).

### Model for a Single-Channel Pneumatic Actuator

The total potential energy of the single-channel phase change actuator upon inflation was expressed from Equation 1 as:

(4) $$\begin{array}{ll} U_{\text{total}} & =\frac{1}{2}E∭\left[{\left(\varepsilon_{\text{circumferential}}\right)}^{2}+{\left(\varepsilon_{\text{axial}}\right)}^{2}+{\left(\varepsilon_{\text{radial}}\right)}^{2}\right]\\ dV-P\left(V-V_{0}\right)+P_{\text{ext}}\left(V-V_{0}\right) \end{array}$$

where the first term represented the strain energy in the soft actuator with *ε*_circumferential_, *ε*_axial_ and *ε*_radial_ being circumferential, axial and radial strain, respectively. The second and third terms represented the work done by the inner and external pressure, respectively. *P* was the inner pressure, and *V* was the volume of the actuator. *P*_ext_ was the pressure exerted by the external environment and *V*_0_ was the initial volume. For a thin wall vessel, *ε*_radial_ was negligible compared to the hoop (circumferential) and axial strain. Then, the Equation 4 above could be rewritten as:

(5) $$U_{\text{total}}=\frac{1}{2}E\int_{0}^{2\pi }\int_{0}^{L}\int_{r-\frac{t}{2}}^{r+\frac{t}{2}}{\left(\frac{r}{r_{0}}-1\right)}^{2}\text{rdrdLd}\theta +\frac{1}{2}E{\left(\frac{L}{L_{0}}-1\right)}^{2}\times 2\pi r\times t\times L-\left(P-P_{\text{ext}}\right)\left(\pi r^{2}L-\pi r_{0}^{2}L_{0}\right)$$

where the first and second terms represented the circumferential stretching energy and axial stretching energy in the soft actuator, respectively. *L*, *r*, and *t* were the actuator’s length, radius and thickness, respectively. *E* denoted Young’s modulus of the soft material. *r*_0_ was the initial radius, and *L*_0_ was the initial length of the soft actuator. The radius and length of the actuator at equilibrium state, *r*_eq_ and *L*_eq_, could be obtained by minimizing the total potential energy, i.e.:

(6) $$\{\begin{array}{c} \frac{dU_{\text{total}}}{dr}=0\\ \frac{dU_{\text{total}}}{dL}=0 \end{array}$$

The predicted value of *r*_eq_ and *L*_eq_ could be obtained by numerically solving Equation 6.

### Responsibility of the Magnetic Phase-Change Actuator

In this section, the required time was studied for the inflation and deflation process separately with different thicknesses, moduli and the contents of Fe_3_O_4_NP, as shown in Figure S17 (Supporting Information). In the inflation process, there were two factors that affect the inflation time, i.e., heating efficiency and strain energy of the composed soft skin (*U*_actuator_ in Equation 1). Figure S17a (Supporting Information) showed the inflation time decreases from 11.4 to 5 s, with the increasing Fe_3_O_4_NP content from 10 to 40 wt.%. This was because the higher Fe_3_O_4_NP content could improve the heating efficiency. Figure S17b (Supporting Information) showed that higher moduli (Ecoflex 50) lead to a longer inflation time (7.4 s), as the stiffer actuator required higher inner pressure to actuate. Figure S17c (Supporting Information) showed the inflation time decreases to 3.7 s with the thickness increase to 300 μm. In this case, increasing the thickness without changing the Fe_3_O_4_NP concentration meant more overall amount of Fe_3_O_4_ NP. Both the heating efficiency and strain energy of the composed skin increased. Therefore, Figure S17c (Supporting Information) implied that the increase in heating efficiency dominated.

In the cooling process, the deflation time mainly depended on the thermal conductivity and the strain energy of the composed soft skin. Figure S17d (Supporting Information) showed the deflation time decreases from 41.8 to 33.5 s when the Fe_3_O_4_NP content increases from 10 to 40 wt.%. This was because the thermal conductivity increases with the Fe_3_O_4_NP content.^[45]^ Figure S17e (Supporting Information) showed a quicker deflation (29.6 s) when the moduli was high (Ecoflex 50), due to the increasing strain energy. Figure S17f (Supporting Information) showed the deflation time was prolonged from 33.5 to 39.6 s when the actuator thickness increased from 100 to 300 μm. This was because actuators with thicker skin were harder to transfer heat. Although thicker skin had higher strain energy, slower heat transfer dominated this competition.

### Work Capacity of the Magnetic Phase-Change Actuator

It is assumed the actuator is applied with a uniform external pressure, *P*_ext_, during the inflation process. The corresponding work capacity (*w*) was work per unit weight, which can be obtained by:^[46]^

(7) $$w=\frac{P_{\text{ext}}\Delta V}{m}=\frac{P_{\text{ext}}\left(\pi r^{2}L-\pi r_{0}^{2}L_{0}\right)}{m}$$

where *m* was the mass of the actuator. *L* and *r* were the length and radius of the actuator, which could be numerically solved by Equations 1,4, and 6. Since the change in length of the actuator was smaller than the change in diameter according to Figure 2c, *L* = *L*_0_ was set and thus the second term in Equation 5 was canceled to simplify the model and rewrite it as:

(8) $$U_{\text{total}}=\frac{1}{2}E\int_{0}^{2\pi }\int_{0}^{L_{0}}\int_{r-\frac{t}{2}}^{r+\frac{t}{2}}{\left(\frac{r}{r_{0}}-1\right)}^{2}\text{rdrdLd}\theta -\left(P-P_{\text{ext}}\right)\left(\pi r^{2}L_{0}-\pi r_{0}^{2}L_{0}\right)$$

Minimizing the total potential energy by $\frac{dU_{\text{total}}}{dr}=0$, the quantitative relation could be obtained between the external pressure, inner pressure and the radius of the actuator upon inflation, i.e., the radius change of the actuator at a given *P* as:

(9) $$r=\frac{4Etr_{0}-2\left(P_{\text{ext}}-P\right)r_{0}^{2}+\sqrt{4E^{2}t^{2}r_{0}^{2}-16Etr_{0}^{3}\left(P_{\text{ext}}-P\right)+4{\left(P_{\text{ext}}-P\right)}^{2}r_{0}^{4}-3E^{2}t^{4}}}{6Et}$$

It is found that Equation 9 could be validated by Figure S18 (Supporting Information). It had to be mentioned that the radial expansion ratio shown in Figure S18 (Supporting Information) was calculated from Equation 9 when assuming the *P*_ext_ = 0. The results indicated that the experimental data and the model calculated value match well.

Furthermore, Equation 7 could be rewritten as:

(10) $$w=\frac{P_{\text{ext}}\left(\pi r^{2}-\pi r_{0}^{2}\right)L_{0}}{m}$$

The corresponding work capacity of the soft actuator could be obtained by:

(11) $$w=\frac{P_{\text{ext}}\left\{\pi \frac{\left[20E^{2}t^{2}r_{0}^{2}+8{\left(P_{\text{ext}}-P\right)}^{2}-32Etr_{0}^{3}\left(P_{\text{ext}}-P\right)-3E^{2}t^{4}+4\left(\left(P_{\text{ext}}-P\right)r_{0}^{2}-2Etr_{0}\right)\sqrt{4E^{2}t^{2}r_{0}^{2}-16Etr_{0}^{3}\left(P_{\text{ext}}-P\right)+4{\left(P_{\text{ext}}-P\right)}^{2}r_{0}^{4}-3E^{2}t^{4}}\right]}{36E^{2}t^{2}}-\pi r_{0}^{2}\right\}L_{0}}{m}$$

### Fabrication of the Bistable Shape Memory Sleeve

The bistable shape memory stent was prepared by a template method. First, a 3D direct laser writing system (Photonic Professional, Nanoscribe GmbH, Germany) was used to print the designed bistable structure, and then polydimethylsiloxane (PDMS, Sylgard 184, Dow Corning, base: curing agent at 10:1 mass ratio) elastomer was used to invert the mold to prepare a bistable structure template. Next, to synthesize the shape-memory materials, first, 1 g of poly(bisphenol A-co-epichlorohydrin) glycidyl end-capped (PBGD, Sigma–Aldrich) was taken and heated in an oven at 70 °C for 20 min to melt. Then 360 μL poly(propylene glycol) bis(2-aminopropyl ether) (Jeffamine D230, Sigma–Aldrich), 300 μL neopentyl glycol diglycidyl ether (NGDE, Sigma–Aldrich) and 0.3 g Fe_3_O_4_NPs were taken to add them into the melting PBGD solution. Next, the mixture solution was shaken and stirred for 10 min for thorough mixing. Then the mixed solution was poured into the PDMS template and placed in an oven at 100 °C for 1.5 h and annealed at 130 °C for 1 h to obtain a shape memory scaffold with a bistable structure. Finally, this bistable was wrapped onto the surface of the phase-change soft actuator and was stuck using super glue (Loctite 401).

### Principle of the Reversible Bistable Stent

The key to realizing reversible anchoring lies in the different response temperatures between the magnetic phase-change actuator and the bistable sleeve by tuning the concentration of Fe_3_O_4_NPs in the SMP matrix material. This is because the Fe_3_O_4_NPs concentration determined at which power level (and what temperature) the phase change actuator or the bistable sleeve was activated. In our case, the magnetic phase-change actuator skin contained fewer heating agents (10% mass ratio to the Ecoflex matrix) such that it was actuated by a deploying power input (P2). Conversely, the bistable sleeve, embedded with more heating agents (40% mass ratio to the SMP matrix), was excited by a retrieving power input (P1). In the inflation and sleeve opening process, both actuator and sleeve were heated, exceeding the transition temperature (the transition temperature of the low-boiling point liquid and shape memory polymer were 34 and 30 °C, respectively), but the inflation force dominated. After removing the input signal, the phase change actuator deflated; meanwhile, the SMP sleeve cooled below the transition temperature and preserved the open state due to the bistable structure. Then, when a retrieving power input (P1) was applied, the retraction deformation of the SMP dominated, leading to the robot recovering to the original state.

### Porcine Coronary Artery Preparation for Ex Vivo Tests

The porcine hearts were used as side products of the animals. The authorities for official food control, consumer protection and veterinary services of the state capital Stuttgart issued the permit and registration number for the usage of these side products for scientific research (registration number: DE 08 111 1008 21). The coronary arteries were cut from the fresh porcine hearts that had been killed and refrigerated under 4 °C within 48 h (Slaughterhouse Ulm, Germany, and Gourmet Compagnie GmbH, Germany). The sliced tissues were washed with phosphate-buffered saline (PBS, pH = 7.4, Gibco, Thermo Fisher Scientific) before tests. Following the experiment, the used animal byproducts were pressure sterilized.

### Statistical Analysis

The length elongation ratio and radial expansion ratio shown in Figure 2c were calculated by $\frac{L}{L_{0}}$ and $\frac{r}{r_{0}}$, respectively, where $L_{0}=10\;\text{mm}$ and $r_{0}=1.5\;\text{mm}$ were the initial value. The volume expansion ratios shown in Figure 2d,e and Figures S7 and S18 (Supporting Information) were calculated by $\frac{L\times r^{2}}{L_{0}\times r_{0}^{2}}$. The output forces of the actuator shown in Figure 2f and Figures S8 and S9 (Supporting Information) were calculated by Equation 2. The remaining experimental data were raw data. The experimental data are presented as mean values ± standard deviation for *n* times measured. In this work, unless otherwise mentioned, the size of this magnetic phase-change actuator was 10 mm × 3 mm (length × diameter). The Microsoft Excel software was utilized to carry out the statistical analysis.

## Supplementary Material

Supplemental Materials

Supplemental Movie 1

Supplemental Movie 2

Supplemental Movie 3

Supplemental Movie 4

Supplemental Movie 5

Supplemental Movie 6

Supplemental Movie 7

Supplemental Movie 8

Supplemental Movie 9

Supplemental Movie 10

## Data Availability

The data that support the findings of this study are available from the corresponding author upon reasonable request.

## References

[1] Chung SE, Dong X, Sitti M (2015). Lab Chip.

[2] Tasoglu S, Diller E, Guven S, Sitti M, Demirci U (2014). Nat Commun.

[3] Li M, Pal A, Aghakhani A, Pena-Francesch A, Sitti M (2022). Nat Rev Mater.

[4] Glick P, Suresh SA, Rufatto D, Cutkosky M, Tolley MT, Parness A (2018). IEEE Robot Autom Lett.

[5] Tang Y, Chi Y, Sun J, Huang TH, Maghsoudi OH, Spence A, Zhao J, Su H, Yin J (2020). Sci Adv.

[6] Villegas D, van Damme M, Vanderborght B, Beyl P, Lefeber D (2012). Adv Robot.

[7] Dobbelsteen JJvd, Lee RA, Noorden Mv, Dankelman J (2012). Med Biol Eng Comput.

[8] Cabrera MS, Oomens CWJ, Baaijens FPT (2017). J Mech Behav Biomed Mater.

[9] Hu W, Alici G (2019). Soft Robot.

[10] Jani JM, Leary M, Subic A, Gibson MA (2014). Mater Des.

[11] Shepherd RF, Ilievski F, Choi W, Morin SA, Stokes AA, Mazzeo AD, Chen X, Wang M, Whitesides GM (2011). Proc Natl Acad Sci USA.

[12] Mirvakili SM, Sim D, Hunter IW, Langer R (2020). Sci Robot.

[13] Mirvakili SM, Hunter IW (2021). Massachusetts Institute of Technology, MA (US) US2021/0355972A1.

[14] Han J, Jiang W, Niu D, Li Y, Zhang Y, Lei B, Liu H, Shi Y, Chen B, Yin L, Liu X, Peng D, Lu B (2019). Adv Intell Syst.

[15] Eristof S, Kim SY, Sanchez-Botero L, Buckner T, Yirmibeşoğlu OD, Kramer-Bottiglio R (2022). Adv Mater.

[16] Yang G-Z, Bellingham J, Dupont PE, Fischer P, Floridi L, Full R, Jacobstein N, Kumar V, McNutt M, Merrifield R, Nelson BJ, Scassellati B, Taddeo M, Taylor R, Veloso M, Wang ZL, Wood R (2018). Sci Robot.

[17] Hirai S, Nagatomo T, Hiraki T, Ishizuka H, Kawahara Y, Miki N (2021). IEEE Robot Autom Lett.

[18] Hu W, Lum GZ, Mastrangeli M, Sitti M (2018). Nature.

[19] Park J-k, Lim KS, Bae I-H, Nam J-P, Cho JH, Choi C, Nah J-W, Jeong MH (2016). J Mech Behav Biomed Mater.

[20] Singh NH, Schneider PA, Moore WS, Ahn SS (2011). Endovascular Surgery Ch. 8.

[21] Beer FP, Johnston JER, Dewolf JT, Mazurek DF (2012). Mechanics of Materials.

[22] Chu H, Hu X, Wang Z, Mu J, Li N, Zhou X, Fang S, Haines CS, Park JW, Qin S, Yuan N (2021). Science.

[23] Haines CS, Lima MD, Li N, Spinks GM, Foroughi J, Madden JDW, Kim SH, Fang S, Andrade MJ, Göktepe F, Göktepe Ö (2014). Science.

[24] Hamiti K, Voda-Besançon A, Roux-Buisson H (1996). Control Eng Pract.

[25] Malakar AK, Choudhury D, Halder B, Paul P, Uddin A, Chakraborty S (2019). J Cell Physiol.

[26] Kennedy SA, Mafeld S, Baerlocher MO, Jaberi A, Rajan DK (2019). J Vasc Interv Radiol.

[27] Klarhöfer M, Csapo B, Balassy C, Szeles JC, Moser E (2001). Magn Reson Med.

[28] Borghi A, Murphy O, Bahmanyar R, McLeod C (2014). J Mater Eng Perform.

[29] Li C, Zhang T, Goldman DI (2013). Science.

[30] Naclerio ND, Karsai A, Murray-Cooper M, Ozkan-Aydin Y, Aydin E, Goldman DI, Hawkes EW (2021). Sci Robot.

[31] Fouillet Y, Parent C, Gropplero G, Davoust L, Achard JL, Revol-Cavalier F, Verplanck N (2017). Proceedings.

[32] Ji X, Liu X, Cacucciolo V, Imboden M, Civet Y, Haitami AE, Cantin 5, Perriard Y, Shea H (2019). Sci Robot.

[33] Wehner M, Truby RL, Fitzgerald DJ, Mosadegh B, Whitesides GM, Lewis JA, Wood RJ (2016). Nature.

[34] Zhao Y, Xuan C, Qian X, Alsaid Y, Hua M, Jin L, He X (2019). Sci Robot.

[35] Jones TJ, Jambon-Puillet E, Marthelot J, Brun PT (2021). Nature.

[36] Lum GZ, Ye Z, Dong X, Marvi H, Erin O, Hu W, Sitti M (2016). Proc Natl Acad Sci USA.

[37] Ren Z, Zhang R, Soon RH, Liu Z, Hu W, Onck PR, Sitti M (2021). Sci Adv.

[38] McKeen LW (2016). Permeability properties of plastics and elastomers.

[39] Noguchi T, Tsumori F (2020). Jpn J Appl Phys.

[40] Huang X, Liu G, Wang X (2012). Adv Mater.

[41] Chen K, Li L (2019). Adv Mater.

[42] Lacroix LM, Malaki RB, Carrey J, Lachaize S, Respaud M, Goya GF, Chaudret B (2009). J Appl Phys.

[43] Davis HC, Kang S, Lee J-H, Shin T-H, Putterman H, Cheon J, Shapiro MG (2020). Biophys J.

[44] Zhang J, Ren Z, Hu W, Soon RH, Yasa IC, Liu Z, Sitti M (2021). Sci Robot.

[45] Chi Q, Ma T, Dong J, Cui Y, Zhang Y, Zhang C, Xu S, Wang X, Lei Q (2017). Sci Rep.

[46] Callen HB (1985). Thermodynamics and an Introduction to Thermostatistics.

