# Supplementary material for: Wireless Miniature Magnetic Phase-Change Soft Actuators
Source: Adv Mater. Author manuscript; Available in PMC 2022 Oct 6. (PMC7613683; doi:10.1002/adma.202204185)
Supplement: Supplemental Materials [file EMS153890-supplement-Supplemental_Materials.pdf]

ADVANCED MATERIALS

Supporting Information

for *Adv. Mater.,* DOI: 10.1002/adma.202204185

Wireless Miniature Magnetic Phase-Change Soft Actuators

Yichao Tang, Mingtong Li, Tianlu Wang, Xiaoguang Dong, Wenqi Hu, and Metin Sitti**

Supporting Information

Wireless Miniature Magnetic Phase-Change Soft Actuator

Yichao Tang, Mingtong Li, Tianlu Wang, Xiaoguang Dong, Wenqi Hu and Metin Sitti

This file includes:

Tables S1 to S2

Figures S1 to S18

Captions for Movies S1 to S11

Table S1. Comparison of the proposed work with the typical phase-change soft actuators works in the literature.

Type	Actuation method	Size	Volumetric strain (%)	Work density (kJ/m^3)
**This work**	Wireless magnetic-RF heating	Diameter: 3 mm<br>Length: 10 mm	7000	3×10^5
**Ref. 9e**	Electronic wire	Diameter: 20 mm<br>Length: 100 mm	900	700
**Ref. 12, 13**	Wireless magnetic-RF heating	Diameter: 16.9 mm<br>Length: 63 mm	20	40

Table S2. The power input parameters of the magnetic-RF heating system.

Type	Input power (W)	Current (A)	Frequency (kHz)
**P1**	1461	200	337
**P2**	4449	500	337
**P3**	7435	750	337

Supplementary Figures

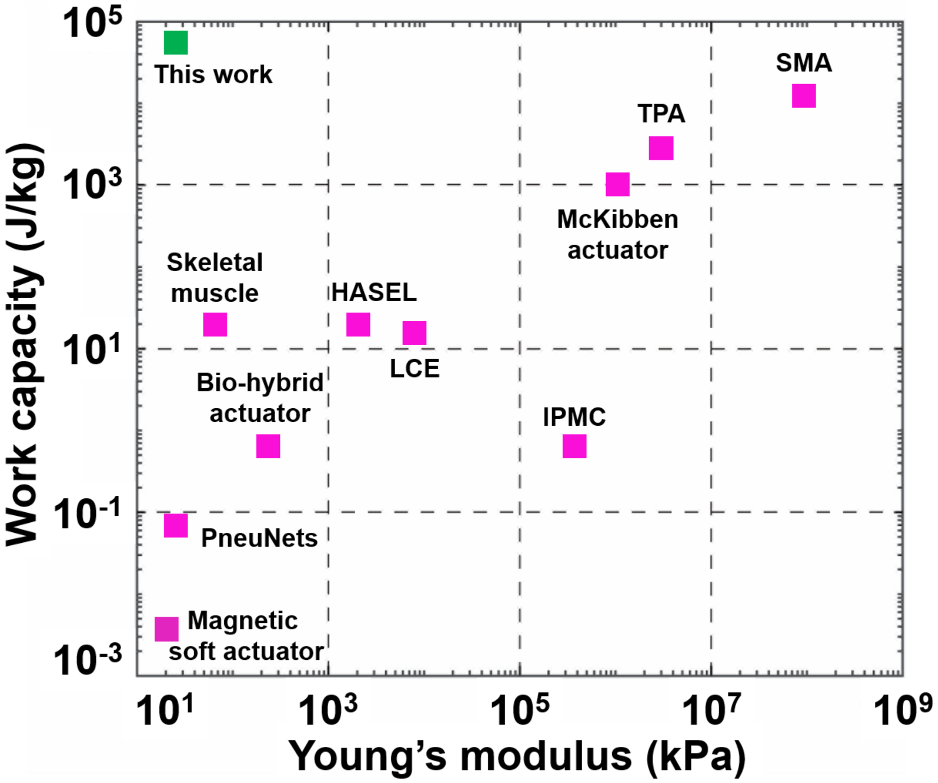

Figure S1. Comparison of different types of soft actuators in terms of the constituent materials modulus and the output work capacity. This includes the currently proposed actuator (this work), Skeletal muscle (Ref. 3b), Bio-hybrid actuator (Ref. 6c), Pneumatic networks (PneuNets, Ref. 6g), Magnetic soft actuator (Ref. 6f), Hydraulically amplified self-healing electrostatic actuator (HASEL, Ref. 6d), Liquid crystal elastomer (LCE, Ref. 6b), Twisted polymer actuator (TPA, Ref. 6h), McKibben actuator (Ref. 6a), Ionic polymer-metal composites (IPMC, Ref. 6e), Shape memory alloy (SMA, Ref. 7a).

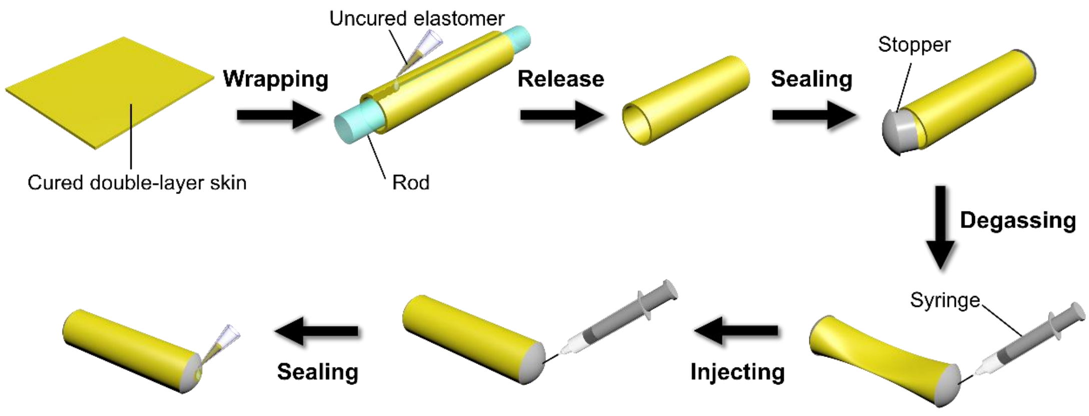

Figure S2. Fabrication schematic of the magnetic phase-change actuator.

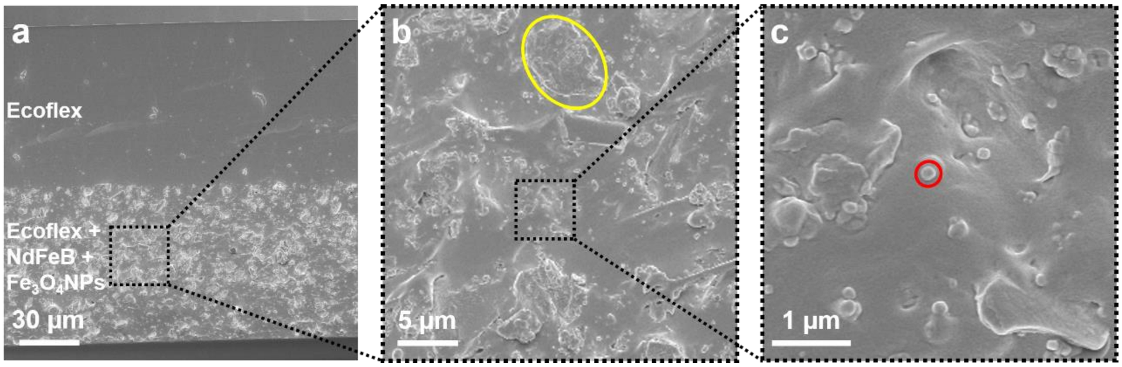

Figure S3. (a) SEM and (b-c) enlarged SEM images of the composed double-layer actuator skin. Note, the part surrounded by the yellow circle in (b) and similar amorphous shapes represent NdFeB microparticles; the part enclosed in the red circle in (c) and similar spheres represent Fe_3O_4NPs.

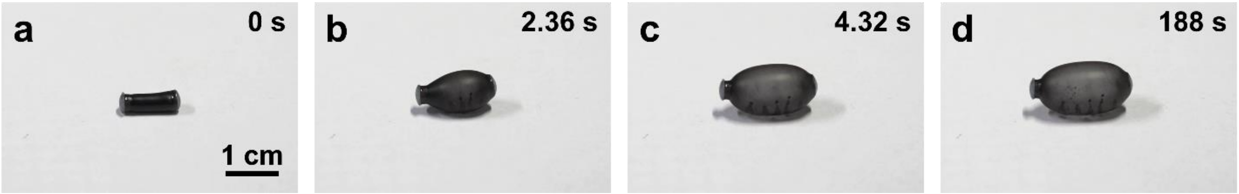

Figure S4. (a-d) The images of the actuation and retention of the phase change actuator under continuous magnetic-RF heating.

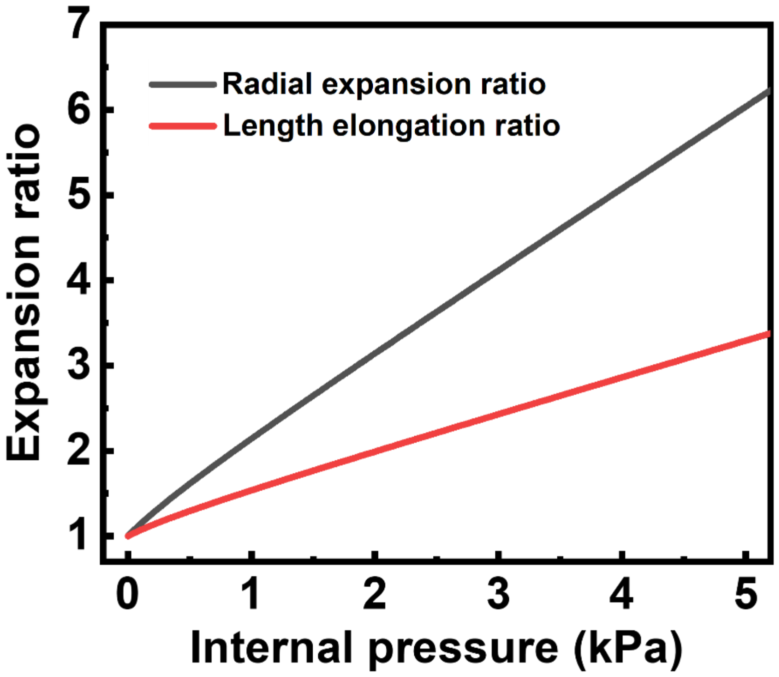

Figure S5. Analytical radial expansion ratio and longitudinal expansion ratio of the magnetic phase-change soft actuator versus the inner pressure.

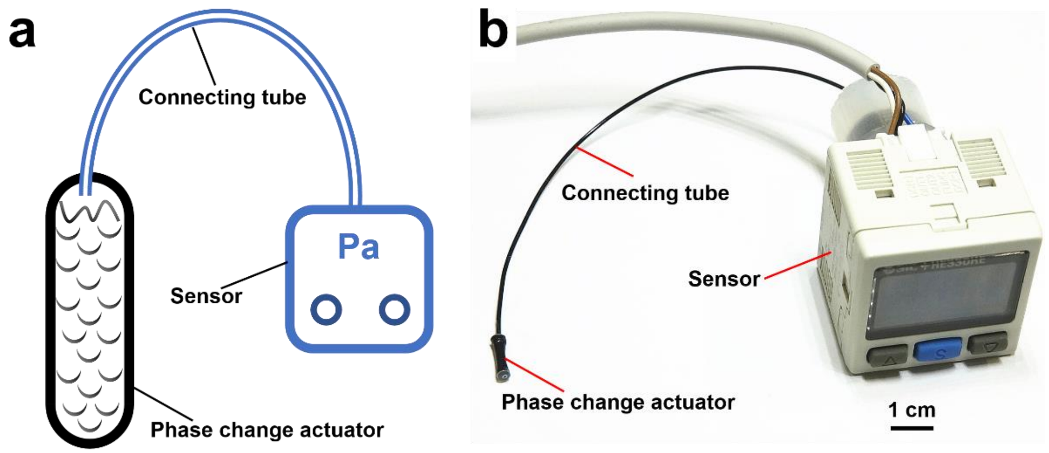

Figure S6. (a) The schematic and (b) image of the setup to measure the internal pressure of the actuator.

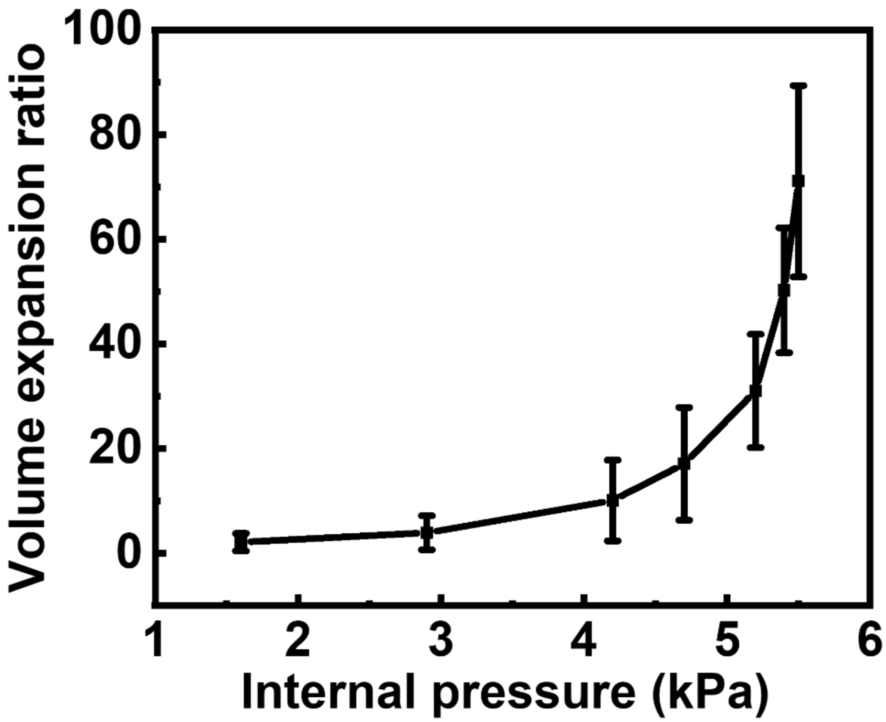

Figure S7. The volume expansion ratio of the magnetic phase-change soft actuator under different internal pressures. The data are presented as mean values ± standard deviation for 3 times measured.

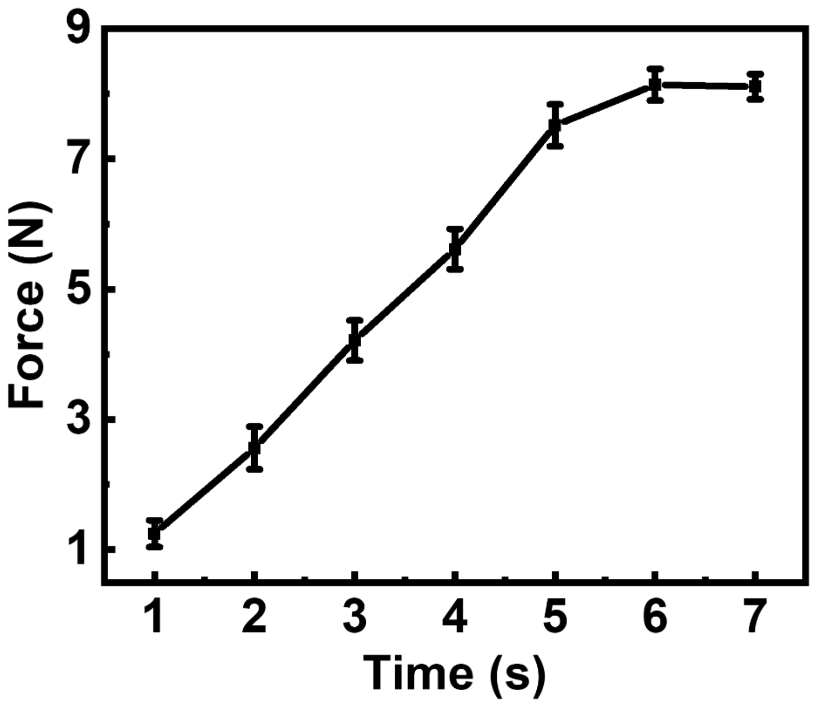

Figure S8. The output force under magnetic-RF heating when confined the magnetic phase-change actuator into a glass tube with a volume of 70.65 mm^3. The data are presented as mean values ± standard deviation for 3 times measured.

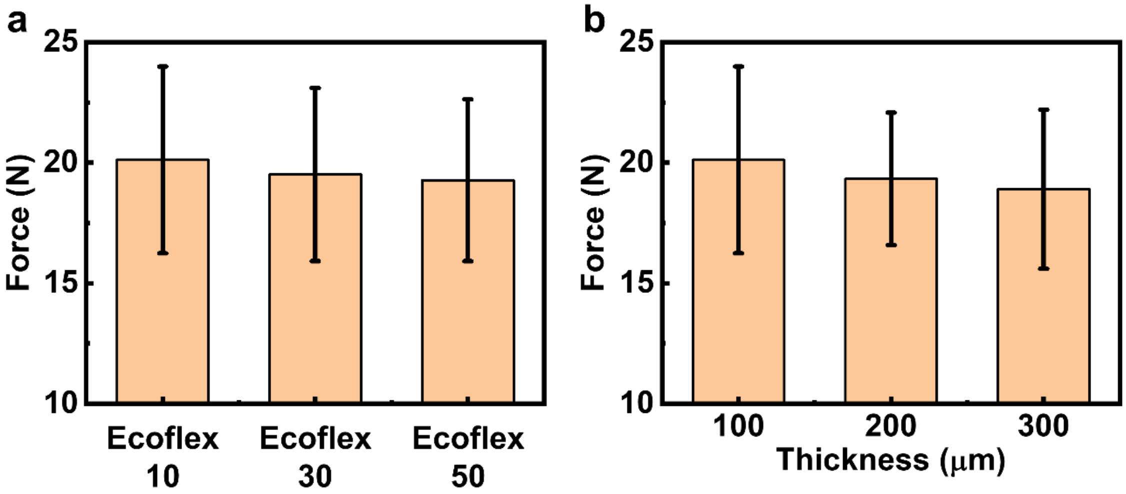

Figure S9. The output force of the magnetic phase-change soft actuator with different (a) composed material stiffness and (b) skin thickness. Note, the volume expansion ratio is fixed at about 10. The data are presented as mean values ± standard deviation for 3 times measured.

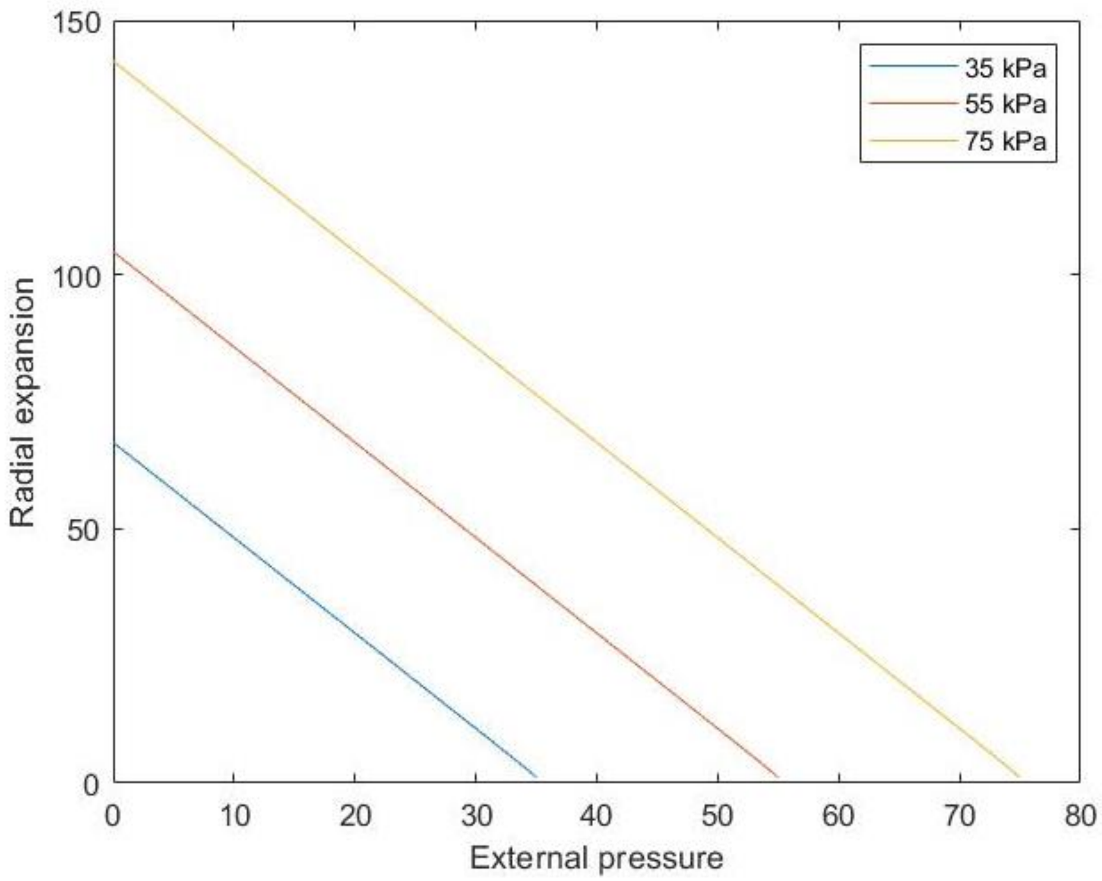

Figure S10. The theoretical radial inflation of the actuator as a function of external pressure with different inner pressure.

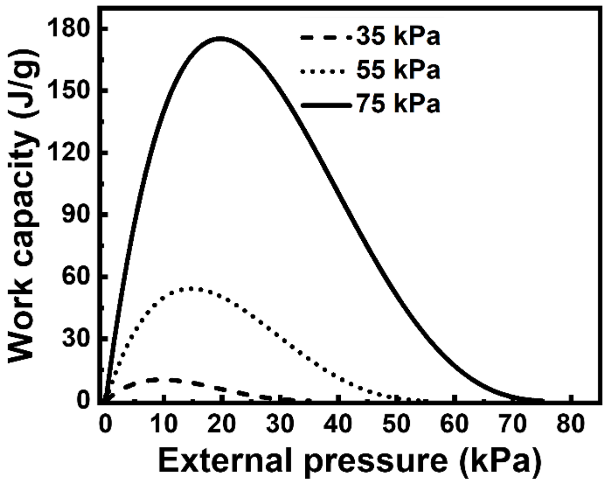

Figure S11. The calculated work capacity under different external pressure.

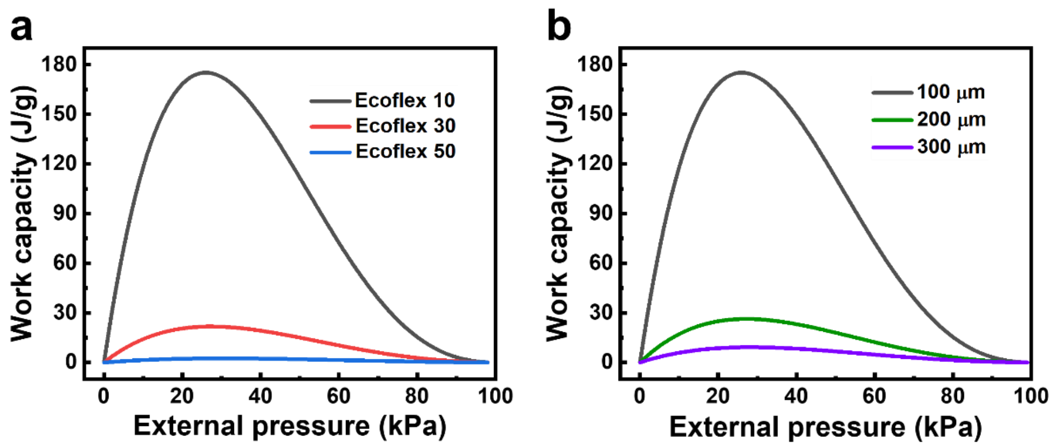

Figure S12. Characterization of work capacity. The calculated work capacities with different (a) moduli and (b) thickness of the composed skin versus the external pressure. Note, the actuator thickness in (a) is 100 μm and Ecoflex 10 is selected in (b).

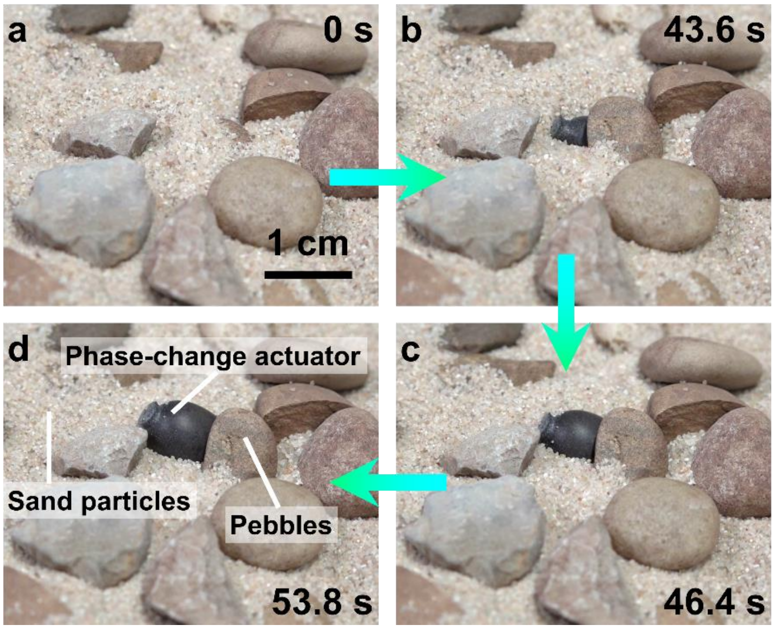

Figure S13. The locomotion of the phase change actuator inside the granular media with sand and pebbles.

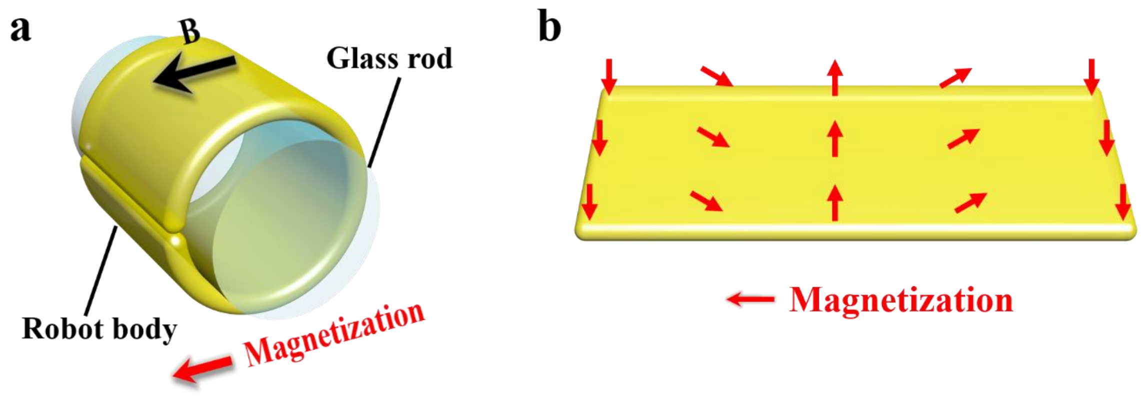

Figure S14. Schematic of the magnetization process. (a) Schematic showing the magnetic phase-change soft actuator body is rolling on the surface of a glass rod and magnetizing with a uniform magnetic field of 1.8 T. (b) Schematic illustrates the corresponding magnetization direction distribution of the actuator body after the magnetization process.

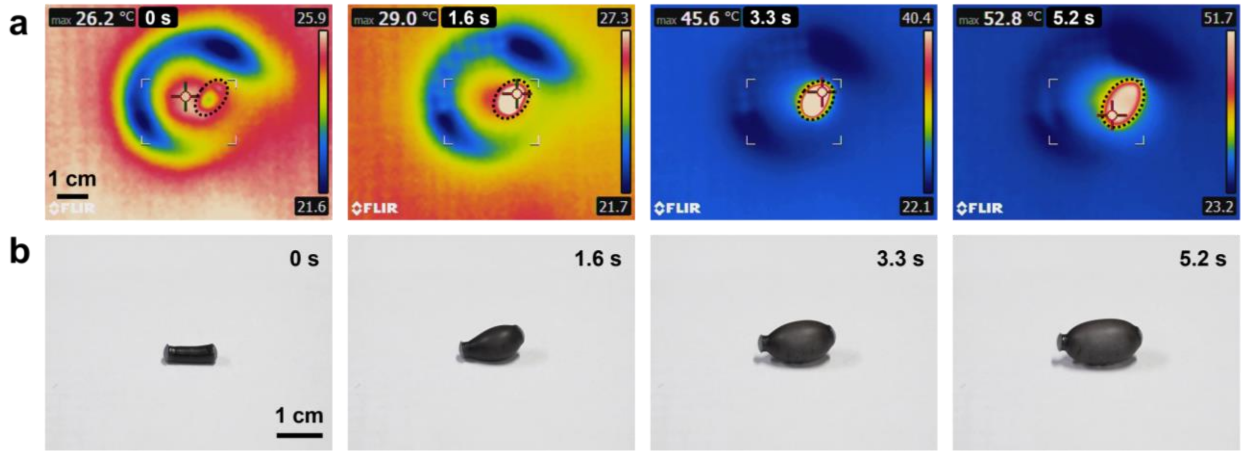

Figure S15. (a) Infrared thermal images and (b) the camera images of the phase change actuator when actuated under magnetic-RF heating. Note, the infrared thermal images shown in (a) were recorded from the top view, and the black dash line represents the position of the phase change actuator.

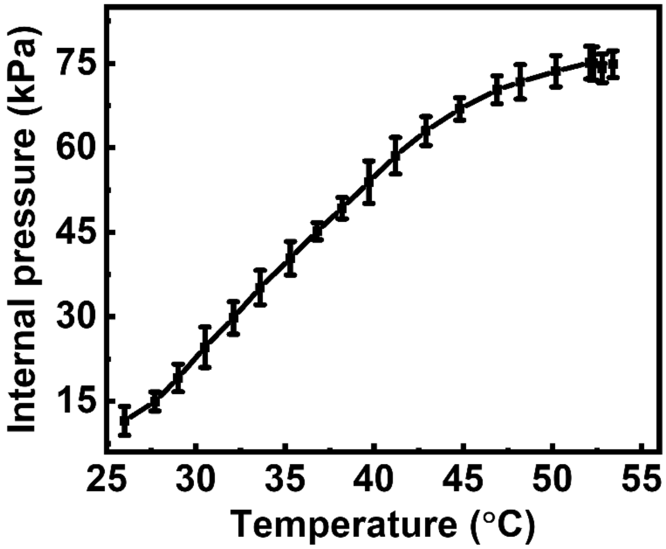

Figure S16. The relationship between the actuation temperature and the internal pressure. The data are presented as mean values ± standard deviation for 3 times measured.

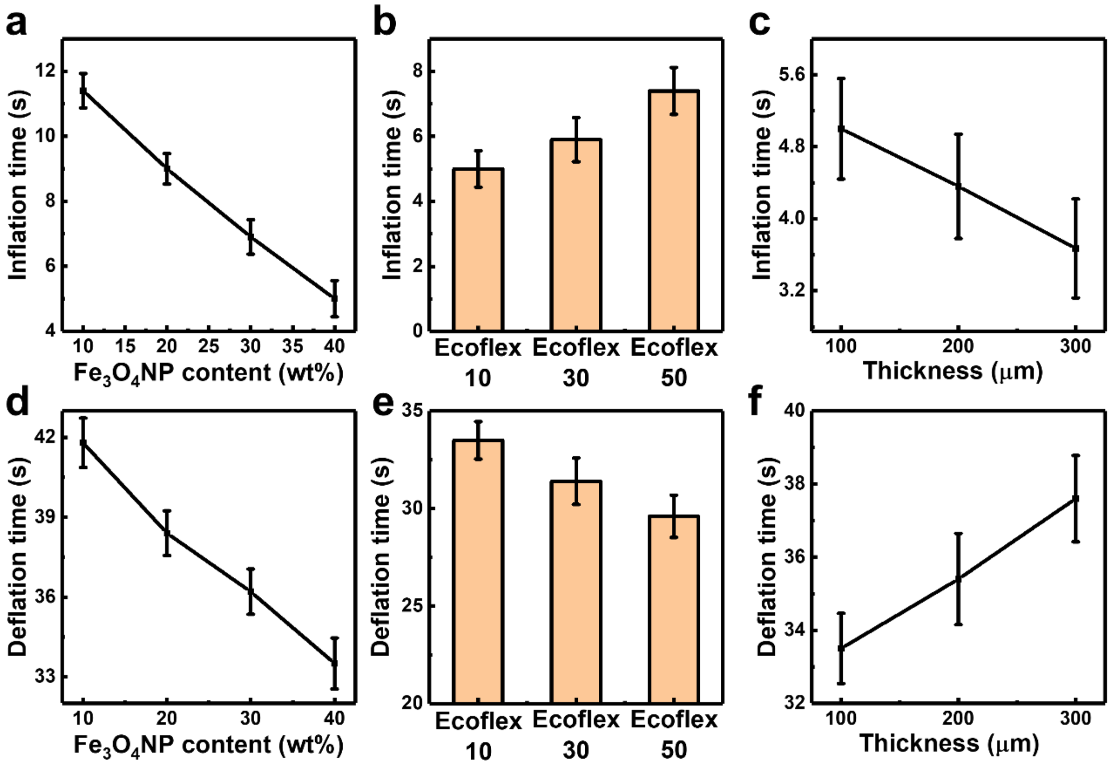

Figure S17. Characterization of actuation response time. Influence of (a,d) Fe_3O_4NP content, (b,e) moduli (Young's modulus of the composed Ecoflex 10, 30 and 50 are 20.4, 43.2, 104 kPa, respectively) and (c,f) skin thickness of the composed materials on the inflation time and deflation time, respectively. The data are presented as mean values ± standard deviation for 3 times measured.

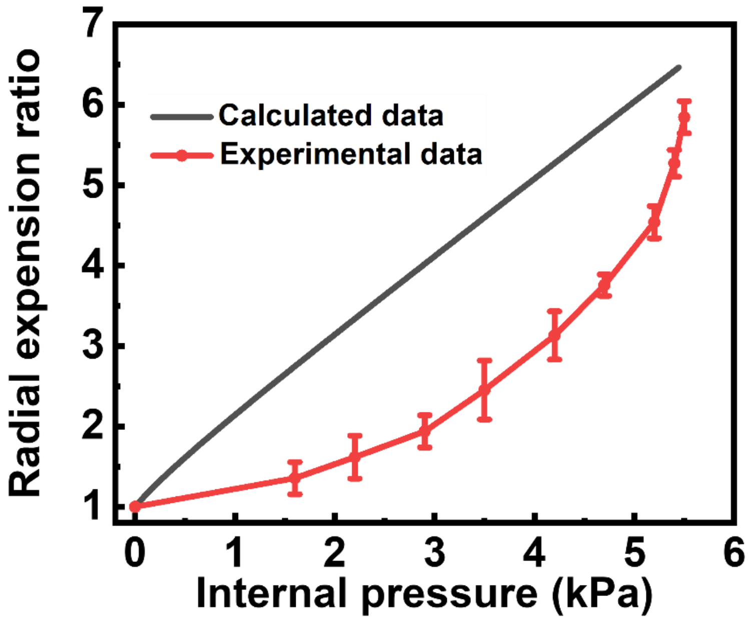

Figure S18. The calculated and experimental data for the body radial inflation ratio of the actuator as a function of the inner pressure. Note, this figure is a combination of Figure 2c and Figure S5. The data are presented as mean values ± standard deviation for 3 times measured.

Supplementary Movies

Movie S1. The inflation process of the magnetic phase-change soft actuator under magnetic-RF heating.

Movie S2. The inflation and retention of the phase-change actuator under continuous magnetic-RF heating.

Movie S3. The inflation process of a cylindrical actuator inside a pig artery.

Movie S4. The magnetic phase-change actuator's locomotion, inflation, and blocking process under a rotating magnetic field and magnetic-RF heating, respectively, inside a glass tube platform.

Movie S5. The operation process of the reversible stent with a controlled power input of the magnetic-RF heating.

Movie S6. The "open" and "close" process of the reversible stent under the controlled magnetic-RF heating inside an Ecoflex soft tube.

Movie S7. The reversible stent opens inside the pig artery under magnetic-RF heating, which was obtained by the ultrasound camera.

Movie S8. The lifting process of the magnetic phase-change actuator inside a granular media by turning the magnetic-RF heating system on and off at regular intervals.

Movie S9. The explosion and jumping behavior of the magnetic phase-change actuator in granular media under magnetic-RF heating.

Movie S10. The locomotion behavior of the phase-change actuator inside the granular media with sand and pebbles.

Movie S11. The bending and expanding behavior of the phase-change actuator under magnetic torques and magnetic-RF heating, respectively.
